# Acquisition of Aneuploidy Provides Increased Fitness during the Evolution of Antifungal Drug Resistance

**DOI:** 10.1371/journal.pgen.1000705

**Published:** 2009-10-30

**Authors:** Anna M. Selmecki, Keely Dulmage, Leah E. Cowen, James B. Anderson, Judith Berman

**Affiliations:** 1 Department of Genetics, Cell Biology and Development, University of Minnesota, Minneapolis, Minnesota, United States of America; 2 Department of Molecular Genetics, University of Toronto, Toronto, Canada; 3 Department of Ecology and Evolutionary Biology, University of Toronto, Mississauga, Ontario, Canada; 4 Department of Microbiology, University of Minnesota, Minneapolis, Minnesota, United States of America; University of California San Francisco, United States of America

## Abstract

The evolution of drug resistance is an important process that affects clinical outcomes. Resistance to fluconazole, the most widely used antifungal, is often associated with acquired aneuploidy. Here we provide a longitudinal study of the prevalence and dynamics of gross chromosomal rearrangements, including aneuploidy, in the presence and absence of fluconazole during a well-controlled *in vitro* evolution experiment using *Candida albicans*, the most prevalent human fungal pathogen. While no aneuploidy was detected in any of the no-drug control populations, in all fluconazole-treated populations analyzed an isochromosome 5L [i(5L)] appeared soon after drug exposure. This isochromosome was associated with increased fitness in the presence of drug and, over time, became fixed in independent populations. In two separate cases, larger supernumerary chromosomes composed of i(5L) attached to an intact chromosome or chromosome fragment formed during exposure to the drug. Other aneuploidies, particularly trisomies of the smaller chromosomes (Chr3–7), appeared throughout the evolution experiment, and the accumulation of multiple aneuploid chromosomes per cell coincided with the highest resistance to fluconazole. Unlike the case in many other organisms, some isolates carrying i(5L) exhibited improved fitness in the presence, as well as in the absence, of fluconazole. The early appearance of aneuploidy is consistent with a model in which *C. albicans* becomes more permissive of chromosome rearrangements and segregation defects in the presence of fluconazole.

## Author Summary

*C. albicans*, the most prevalent human fungal pathogen, acquires resistance to fluconazole by genetic alterations that often include changes in the number of chromosomes or chromosome arms (aneuploidy). Here we demonstrate that chromosomal rearrangements resulting in increased gene dosage are the predominant means of acquired resistance to the antifungal drug fluconazole in replicated experimental populations of *C. albicans*. A specific aneuploidy, isochromosome 5L, which is composed of two copies of the left arm of Chr5, occurs with high frequency and is detectable soon after fluconazole exposure. The early appearance of aneuploidy in some populations is consistent with a model in which *C. albicans* becomes more permissive of chromosome rearrangements and segregation defects in the presence of fluconazole. The results presented here indicate that the *C. albicans* genome is highly plastic and imply that exposure to an antifungal drug induces genome reorganization events, some of which provide a fitness advantage in the presence of drug.

## Introduction

*Candida albicans* is the most prevalent fungal pathogen of humans and is commonly treated with fluconazole because of its low toxicity, low cost and oral availability. The acquisition of drug resistance is an evolutionary process that occurs because antimicrobials rarely kill an entire population [1]. The survivors are subject to strong natural selection for resistant phenotypes in the presence of a drug. The increasing use of prolonged courses of fungistatic antifungal therapies increases the incidence of acquired antifungal drug resistance (reviewed in [2]–[4]). Fluconazole is especially prone to result in resistance as it is fungistatic, not fungicidal and the effective size of surviving populations is large. Furthermore, resistant subpopulations appear to be maintained in the host, since previously treated patients have a higher incidence of resistance to subsequent fluconazole treatments [5].

The evolution of drug resistance depends on phenotypic variability, and the ultimate source of that variability has been considered to be mutations that alter gene expression or protein activities (e.g., [6]). Recent studies indicate that copy number variation (CNV), including short segmental CNV and whole chromosome aneuploidy, are important contributors to genetic variability in human diseases, as well as to the acquisition of resistance to chemotherapeutic agents by tumor cells [7],[8]. Indeed, in *S. cerevisiae*, the increased frequency of CNV and aneuploidy over point mutations during experimental evolution is an indication of the fitness benefit these mutations convey [9]. Fungi exposed to antifungal agents also exhibit high levels of aneuploidy [10]–[13].

In addition to genetic changes in DNA sequence and/or copy number, reproductive output, used to estimate fitness, is an important component of a resistance phenotype [14]. The ability of a pathogen with an altered genotype to survive in the presence and in the absence of drug greatly influences the degree to which that genotype will proliferate in the population and the degree to which it can be targeted if drug regimens are changed. However, the fitness effect of CNVs in the presence and absence of drugs has not been studied systematically. In particular, the dynamics of aneuploidy acquisition, fixation and change in a population over the course of physiologically relevant drug treatment have not been examined.

Molecular mechanisms of resistance to fluconazole are well documented and include alterations of two general processes. First, lanosterol 14-alpha-demethylase, which catalyzes a critical step in the ergosterol biosynthesis pathway (encoded by *ERG11*) is the target of the azole drugs and alterations in this enzyme structure or increases in the level of the protein confer resistance (reviewed in [4]). Second, drug efflux via ABC transporters (encoded by *CDR1* and *CDR2*) or via the major facilitator superfamily efflux pump (encoded by *MDR1*) decreases effective intracellular drug levels, allowing cells to survive in the presence of higher extracellular drug concentrations. Increased activity of these processes can be achieved through point mutations in genes encoding the proteins [15] or in transcription factors that regulate them [16]–[18]. Expression levels [19] or physical copy numbers of these genes can also be increased via genome rearrangements such as whole chromosome and segmental aneuploidies [11],[20].

Alteration in gene copy number is a major mechanism for environmental adaptation of asexual yeast populations [21],[22]. In *C. albicans*, karyotype variability appears in many clinical isolates [23] as well as in some laboratory strains [24],[25]. Aneuploidy is especially common in fluconazole resistant (Flu^R^) strains: approximately 50% of Flu^R^ strains carried a whole chromosome or segmental aneuploidy, while only 10% of fluconazole sensitive (Flu^S^) strains exhibit any type of aneuploidy [11]. One specific segmental aneuploidy, i(5L) (an isochromosome composed of two identical chromosome arms (Chr5L) flanking a centromere), increases gene copy numbers of Chr5L at least 2 fold relative to Chr5R, and appears in >20% of drug resistant strains (12/57 strains analyzed) [11], and A.S. and J.B., unpublished data). The appearance of i(5L) is highly correlated with the appearance of increased Flu^R^, and this Flu^R^ is primarily due to the presence of two genes on Chr5L that contribute additively and independently to Flu^R^ [20]: *ERG11* is located ∼150 kb from the left telomere and *TAC1*, a transcription factor that activates *CDR1* and *CDR2* expression [17],[26], is located ∼48 kb from the centromere. Despite the high prevalence of CNVs in Flu^R^ isolates, no systematic characterization of CNV dynamics has been performed on isogenic *C. albicans* strains throughout the course of fluconazole treatment.

The evolution of drug resistance has been studied by following patient isolates over time. These studies are useful because they provide information about the evolutionary pressures occurring in the patient. For example, data from isolates taken from a patient that acquired Flu^R^ have revealed the homozygosis of point mutations in *ERG11* [27] and in *TAC1* [26]. We recently found that i(5L) was acquired twice, in two genetically distinct bloodstream isolates from an individual patient, and that its appearance correlated with increases in the fluconazole minimal inhibitory concentration (MIC) [20], indicating that active changes in chromosome copy number are an important mechanism for the evolution of antifungal resistance in the clinical setting. However, studies of chronological patient samples are limited because the consecutive events that led to establishment of a specific strain are only inferred [28].

Experimental evolution permits more direct observation of the dynamics of genetic and genomic changes in identical starting populations exposed to a known selective pressure. Multiple, parallel experiments can be performed with controlled conditions of population size and selection pressure in an environment which facilitates sampling of the population throughout the evolutionary process. In addition, they permit analysis of the fitness consequences of those genetic changes and of the frequency of a given genomic change in the population. Parallel evolution of similar genetic changes under identical selection conditions provides strong evidence that these mutations provide an adaptive advantage [29]. For example, evolution of *E. coli* for 20,000 generations resulted in strains with similar expression profiles [30]. Experimental evolution of haploid and/or diploid *S. cerevisiae* in limiting nutrient conditions led to amplification of chromosome regions between highly similar genetic loci [21],[31],[32], as well as reproducible segmental aneuploidies that were specific to the selection environment [29]. In addition, one diploid strain gave rise to trisomy of three whole chromosomes [29].

In the presence of drug, cells that acquire drug resistance are generally more fit than the ancestral strain. In the absence of drug, the relative fitness of these two strains varies [6],[33]. In a previous study, Cowen et al (2000) performed experimental evolution of a single drug sensitive *C. albicans* strain, T118, a strain isolated from an oral swab from a patient that was HIV positive. They used a single colony to seed a liquid culture for one round of overnight growth and then divided this culture into 12 independent T118-derived populations: 6 in the absence of drug (N1–N6) and 6 in the presence of drug (fluconazole, D7–D12). Serial cultures were propagated for ∼330 generations of growth. Each time a culture grew, the amount of fluconazole added to each population was double the MIC. Three independent drug-treated populations acquired very high levels of Flu^R^ and we previously found that i(5L) was present in these three populations (D9-330, D11-330, and D12-165) [11].

Here we studied the evolutionary dynamics of aneuploidy in these twelve batch culture lineages by performing genetic sampling throughout the entire evolution experiment as well as of individuals within mixed subpopulations at times when gross chromosomal rearrangements arose. We performed contour-clamped homogeneous electric field (CHEF) karyotype analysis followed by Southern hybridization to detect gross chromosomal rearrangements, along with comparative genome hybridization (CGH) analysis to detect alterations in chromosome copy number. In addition, we determined the frequency of genome changes by analyzing multiple clones from many of the populations. Following the evolution of initially isogenic populations enabled us to address several questions about the evolution of aneuploidy in *C. albicans*: how early did aneuploidy arise in these populations? What were the dynamics of aneuploid chromosome acquisition and loss in the presence and absence of drug selection? Did specific aneuploidies arise with similar dynamics in different populations? Furthermore, we performed experiments to ask about the fitness effects of the aneuploidies in the presence and absence of fluconazole. We found no evidence for aneuploidy or karyotype changes in the untreated populations or parental strain at any time point. Nonetheless, we found that two specific aneuploid chromosomes, i(5L) and trisomy of Chr7, were detectable within the first drug-exposed passage of all three populations that became highly Flu^R^. Furthermore, additional aneuploidies appeared and disappeared over the course of the experimental evolution. Interestingly, in most cases these aneuploid cells, which carried almost 20% more total DNA content than the progenitor cells, had a clear growth advantage in the presence of fluconazole. Thus, exposure to an antifungal drug led to positive selection of specific aneuploidies and gross chromosomal rearrangements.

## Results

### Karyotype changes are present in early populations that ultimately evolve high levels of Flu^R^

**Figure 1:**
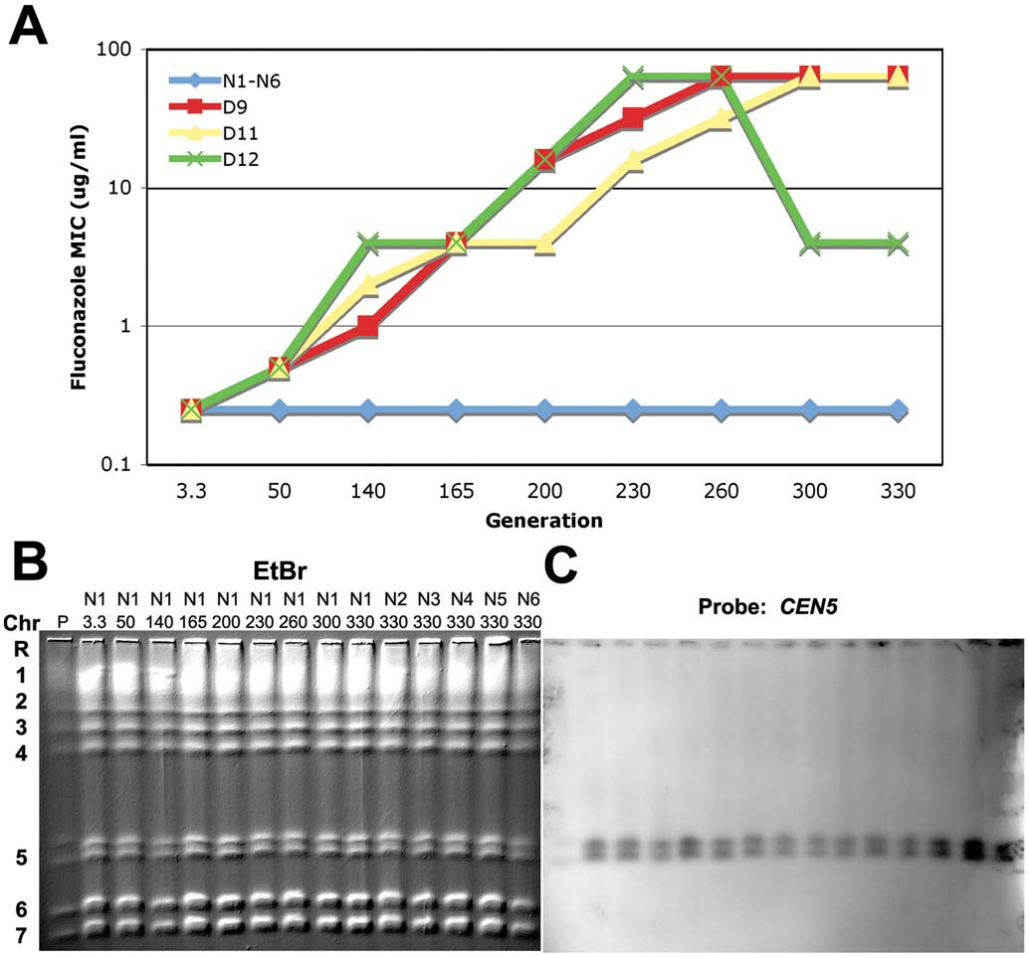
Drug resistance profiles throughout evolution. (A) Populations evolved with fluconazole (D9, D11, and D12) adapted to the drug and showed increased fluconazole MIC [6]. Populations evolved without fluconazole (N1–N6) show no change in MIC and (B) maintain karyotype stability. Whole chromosomes were separated by CHEF, stained with Ethidium Bromide (Left) and (C) subjected to Southern hybridization with a *CEN5* probe. Lane P, the progenitor strain karyotype.

To follow genome dynamics during the evolution of Flu^R^ under controlled experimental conditions, we analyzed three independent fluconazole treated populations (D9, D11 and D12) that developed very high levels of Flu^R^ (MIC peaks of ∼96 µg/ml) ([6] and Figure 1A). We also analyzed six non-drug treated populations derived from the same progenitor strain. In a previous study, we detected i(5L) and other aneuploidies in samples with the highest MIC values from populations that had undergone an estimated 330 or 165 doublings (D9-330, D11-330 and D12-165) [11]. The i(5L) in the tested D9 and D11 samples co-migrated with Chr7 (∼950 kb), the size expected (∼945 kb) for independent i(5L) composed of two copies of Chr5L plus *CEN5*. In the D12-165 sample, the extra Chr5L sequences were found in a much larger band that co-migrated with Chr2 (∼2.2 Mb). Southern analysis demonstrated that this large band was composed of an intact i(5L) attached through a telomere-telomere fusion to the left telomere of an intact copy of Chr5 to form an attached isochromosome 5L (att-i(5L)) [11]. While i(5L) was detected in all three populations, the dynamics of the appearance of i(5L) and other aneuploidies was not known. We did not know when it had arisen and whether or not it had become fixed in the population. Furthermore, while strains containing i(5L) often carried other aneuploid chromosomes as well, it was not clear if those other aneuploidies were more or less stable than the i(5L) in the populations.

**Figure 2:**
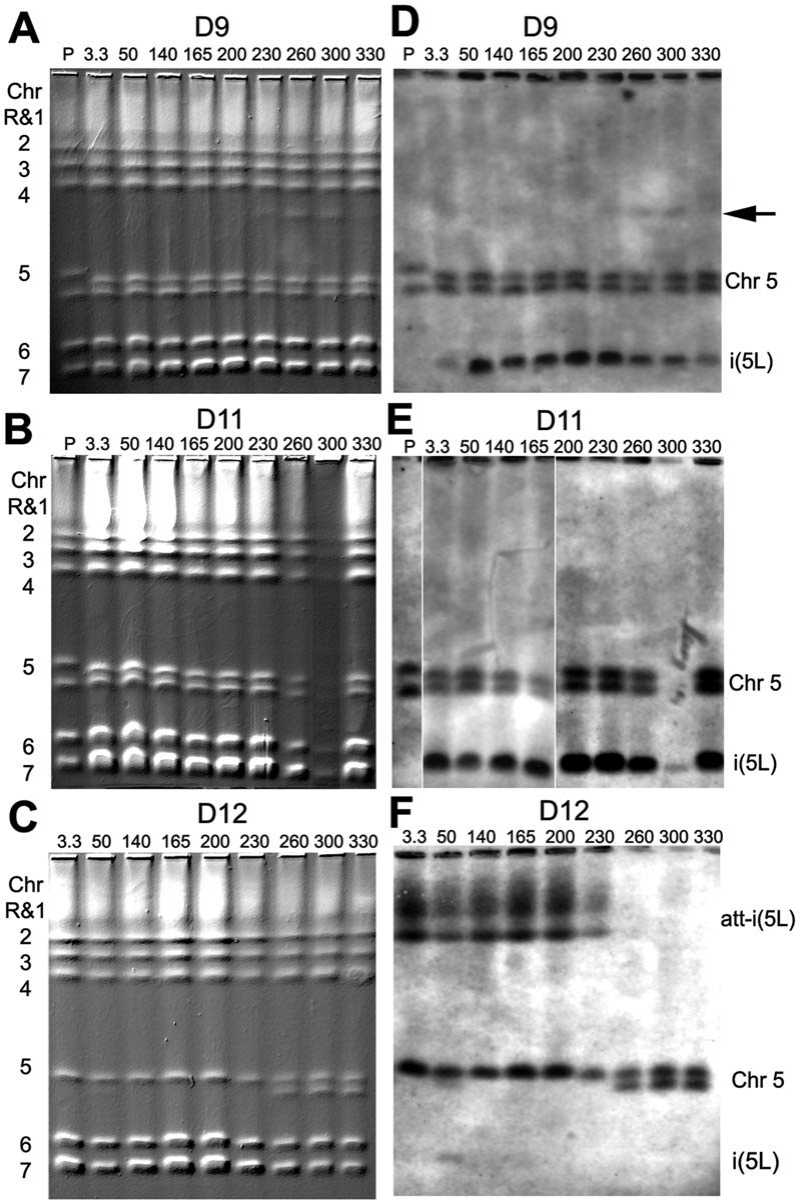
Rapid acquisition of i(5L) occurred in all fluconazole treated populations. (A–C) Whole chromosome CHEF gel analysis of populations D9, D11, and D12 at generations ∼3.3, 50, 140, 165, 200, 230, 260, 300, and 330. (D–F) Southern blot analysis of the CHEF gels using a *CEN5* probe identified both Chr5 homologs as well as either independent i(5L) or att-i(5L) at generation 3.3 in all three populations. The i(5L) aneuploidy was detected in all subsequent generations of D9 and D11, while the att-i(5L) was lost in D12 after generation D12-260. The *CEN5* probe also hybridized to a novel band at ∼1.5 Mb in population D9 (black arrow).

To address questions concerning the dynamics of genome change in these populations, we analyzed the untreated populations (N1 through N6) and all of the time points from the three drug treated populations (D9, D11, and D12) on CHEF karyotype gels stained with EtBr (Figure 1B and Figure 2A–2C), and then analyzed them by Southern hybridization with a *CEN5* probe (Figure 1C and Figure 2D–2F), which detects the i(5L) that co-migrates with Chr7 [11]. In D9, the EtBr-stained karyotypes of early cultures appeared generally unchanged, other than a minor change in Chr5 Major Repeat Sequence (MRS) length (A.S. and J.B., unpublished data) and the appearance of a new band below Chr4 in three later populations (Figure 2D, arrow, discussed below). Surprisingly, Southern analysis revealed a band corresponding to the size of i(5L), in all the evolved cultures, including the ∼3.3 generation isolate. However, the intensity of hybridization in D9-3.3 was lower than in the other generations (Figure 2D). Similar results were seen in population D11, except that hybridization of the *CEN5* probe to i(5L) was stronger than in D9-3.3 (Figure 2E). In the D12 CHEF karyotypes, the acquisition of the att-i(5L) also was evident within the earliest ∼3.3 generation isolate (Figure 2E and 2F) and the shorter Chr5 homolog (*MTL*α, data not shown) ‘disappeared’ at the same time. Importantly, this att-i(5L) was composed of 3 copies of the *MTL*α copy of Chr5L [11]. In contrast, the i(5L) in D9-3.3 and D11-3.3 were composed of 2 copies of the *MTL* ***a*** copy of Chr5L, indicating that the i(5L) in D12 arose independently from the i(5L) found in D9 and D11 populations. At later generations 260, 300 and 330, the att-i5L ‘disappeared’ and the *MTL*α homolog of Chr5 ‘reappeared’. This is consistent with the idea that two events occurred: an i(5L) formed from the *MTL*α homolog of Chr5L and it also became attached to the shorter homolog of Chr5 to form the att-i(5L). Later in the evolution of the culture, the i(5L) portion of the att-i(5L) was lost. Thus, in all three cultures that evolved high levels of Flu^R^, either i(5L) or att-i(5L) was detectable at the earliest sampling of the cultures.

Importantly, neither i(5L) nor any other gross chromosomal rearrangement was detected in the progenitor T118 or in populations that were not treated with drug (Figure 1B and 1C and data not shown). Furthermore, i(5L) was not detected in early time points from drug-treated populations that did not develop high levels of Flu^R^ (D7, D8 and D10, data not shown). In addition, populations of T118 progenitor cells plated on fluconazole concentrations do not contain drug resistant colonies (data not shown). Taken together with the appearance of two different forms of i(5L) generated from different *MTL* alleles, this suggests that the different forms of i(5L) either arose late during growth of the original progenitor colony used to seed all 12 cultures or that the two different isochromosomes arose within the first 24 hours of drug exposure. In either case, growth in fluconazole was selective for the i(5L).

**Figure 3:**
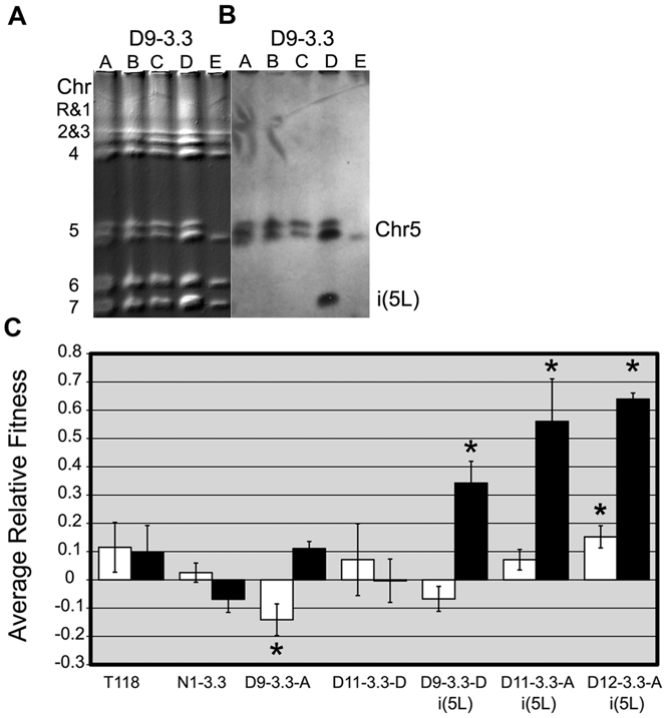
Clones at generation 3.3 with i(5L) have a significant fitness advantage in the presence of fluconazole. (A) Karyotype analysis of five D9-3.3 single colonies by CHEF followed by (B) Southern hybridization to a *CEN5* probe. Genetic heterogeneity and clonal interference exists: 3 clones appear diploid, one clone has i(5L), and one clone is homozygous for Chr5. (C) Fitness competitions were performed, as described in the Materials and Methods, either in the absence (white bars) or presence (black bars) of fluconazole. Each clone was competed against the same *NAT1*-marked progenitor strain and the average relative fitness was determined. Error bars represent the standard deviation of 3 replicate experiments. All three clones with i(5L) had significantly increased fitness in the presence of 0.5 µg/ml fluconazole (asterisks, paired t-test, p≤0.05), with no disadvantage in the absence of the drug.

While only semi-quantitative, Southern analysis of CHEF gels suggested that i(5L) may not be present in all cells in some of the populations such as D9-3.3. To address this issue more directly, we analyzed individual colonies from a number of the time points, to ask if the i(5L) was present in each of the progenitor cells that gave rise to the colonies. Again, CHEF karyotype gels were first analyzed with EtBr and were then subjected to Southern analysis using the *CEN5* probe. An example of the results for colonies derived from the D9-3.3 population is shown (Figure 3). Similar analyses were performed for clones from other populations (Figures S1, S2, S3) and the results are summarized in Table 1.

**Table 1:** Percent of cells in a population with i(5L).

Population	Number of Single colonies with i(5L)	Total Number of colonies analyzed	Percent of Population with i(5L)
D9-3.3	3	16	19%
D9-140	6	6	100
D9-200	6	6	100
D9-260	9	9	100
D11-3.3	15	16	94%
D11-50	11	15	73
D11-140	3	6	50
D11-200	6	6	100
D11-260	6	6	100
D11-330	6	6	100
D12-3.3	15	15	100%
D12-50	10	10	100
D12-230	15	15	100
D12-260	3	15	20

As expected for a mixed population of adaptive mutants, 3 of 16 (19%) of the D9-3.3 clones (individual colonies) contained the i(5L). By the next time point tested (D9-140) all clones carried the i(5L), indicating that it had become fixed in the population. Of note, there was heterogeneity in the genome structures of different clones. For example, the *MTL*α homolog of Chr5 was lost in clone D9-3.3-E (Figure 3A and 3B and data not shown).

Despite the higher proportion of i5L at the D11-3.3 time point, i(5L) did not become fixed as rapidly in this population. After 3.3 doublings, 94% (15 out of 16 clones) contained i(5L); after 50 doublings i(5L) was detected in 73% of the population and by 140 doublings the number decreased to 50% of the clones tested. From 200 doublings on, all clones included an i(5L), suggesting that it had swept the population after 200 doublings.

In D12-3.3 and D12-50, the att-i(5L) was present in all clones analyzed, suggesting that it appeared early after drug exposure. Although CHEF analysis detected a small amount of independent i(5L) (not attached) in population D12-50 (Figure 2F), it was not detectable in any of the 10 clones analyzed. Interestingly, the loss of att-i(5L) appears to have been abrupt as well: it was present in all of the D12-230 clones (15/15 colonies), but only in 20% of the D12-260 clones (3/15 colonies).

### Frequency of i(5L) correlates with fitness levels

The early appearance of i(5L) in all three populations was surprising and could be due to several possible mechanisms. First, it is possible that a subpopulation of the progenitor cells contained i(5L) and that their increased fitness in the presence of drug caused them to be rapidly selected [34]. We consider this unlikely because no i(5L) was detected in early cultures from strains D7, D8 and D10 under drug selection. In the D8 population, i(5L) never appeared and in D7 and D10, which acquired transient peaks of Flu^R^ (MIC ∼4 to 8), i(5L) appeared only in the peak populations (A.M.S., data not shown). Furthermore, since two types of i(5L) (attached *MTL*α/α/α and independent *MTL* ***a/a***) appeared in independent populations, both types would have had to be present in a few cells in the progenitor population and thus each population would have been heterogeneous for different types of i(5L).

Second, it is possible that each i(5L) formed during the 24-hour period of fluconazole stress. Once formed, i(5L) could sweep through the populations if it provided very strong selective advantage in the presence of drug, such that clones that had acquired it out-competed clones within the population that had not acquired it. To ask if this was the case, we compared the fitness of clones with or without i(5L) by directly competing each clone with the drug sensitive ancestor (T118), in the presence and absence of fluconazole, by measuring the reproductive output of each competitor in the final mixture (Figure 3C). In the presence of fluconazole, all clones at generation ∼3.3 that contained an i(5L) exhibited significantly increased fitness relative to the clones that had not acquired i(5L): D9-3.3-D, D11-3.3-A and D12-3.3-A, were 24%, 46% and 54% more fit than the progenitor, respectively. In contrast, the non-i(5L) clones (D9-3.3-A and D11-3.3-D), and the non-drug treated strain (N1-3.3) exhibited fitness levels that were not significantly different from the progenitor in the presence of drug. This indicates that i(5L) confers a strong selective advantage in the presence of the drug. Furthermore, the growth rate and maximum cell density achieved by strains containing i(5L) was much higher than that of sibling strains lacking i(5L) (data not shown). Modeling of cell numbers indicates that if i(5L) arose within the first 2 hours of fluconazole exposure, it could have reached >0.2% of the population within the first growth cycle. Thus, selective advantage alone during 24 hours in fluconazole cannot explain how i(5L) reached levels of 15–100% of the population of ∼1×10∧6 cells. Importantly, cells were stored in the selective growth medium (plus glycerol) immediately following the experiment. We cannot rule out the possibility that resuscitation of those strains may have involved some additional selection that allowed cells containing i(5L) to reach higher proportions of the population.

A third mechanism by which i(5L) may have become a larger proportion of the population is during subsequent non-selective propagation of the strains. Such propagation was necessary for transfer of the strains between labs and for growth and analysis of single colonies for CHEF analysis. We estimate that cells underwent ∼50–70 divisions in the absence of drug prior to CHEF analysis. In fitness assays conducted under the conditions used for the evolution experiment (RPMI medium with or without drug), the D9-3.3, D11-3.3, and D12-3.3 clones that contained the i(5L) had no significant reduction in fitness relative to the progenitor in the absence of drug (Figure 3C); furthermore, D12-3.3 had a significant increase in fitness relative to the progenitor in the absence of fluconazole and D11 consistently exhibited a slight increase in fitness under these conditions. Thus the D12-3.3 population could have easily accumulated more cells carrying i(5L) during the nonselective growth necessary for CHEF gel analysis. This may be the case for D11-3.3 as well. Interestingly, the proportion of cells containing i(5L) in the ‘3.3’ populations reflects the relative fitness advantage of those strains under non-selective conditions. Thus, we conclude that i(5L) arose early in all three populations and reached appreciable proportions of the population during growth in the first cycle of exposure to fluconazole, but that the very high levels of i(5L) found in the populations analyzed is due to that selection plus some additional selective advantage under no drug conditions.

### Multiple aneuploidies are evident in drug-evolved populations

Comparative Genome Hybridization (CGH) performed on a microarray platform that includes probes covering most ORFs in the genome provides a comprehensive view of copy number changes. We used CGH to analyze D9, D11 and D12 populations as well as a number of single colony clones that previously had been analyzed by CHEF karyotype gels and Southern hybridization (e.g., Figure 3, Table 1). In general, strains carrying the ∼945 kb i(5L) on CHEF gels also carried two extra copies of Chr5L, but there were exceptions. In D11-200, -260 and -330, there were >5 copies of Chr5L, suggesting that they contained two copies of i(5L) (4 copies of Chr5L) in addition to the normal two copies on intact Chr5 homologs (Figure S7).

**Figure 4:**
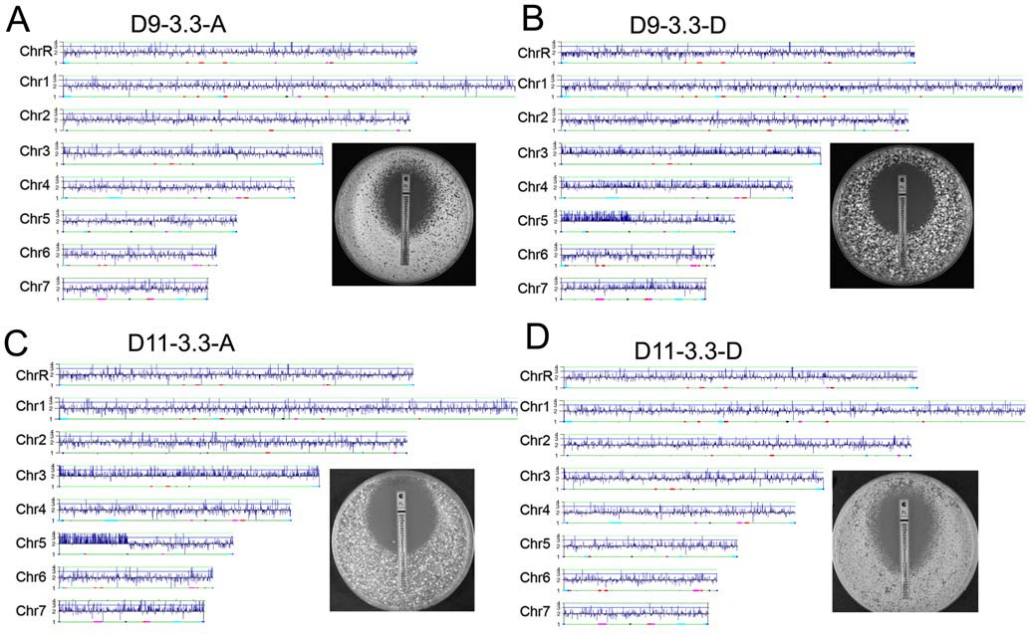
Rapid acquisition of i(5L) coincided with multiple whole chromosome aneuploidies. CGH analysis of single colonies from populations D9-3.3 and D11-3.3 found that clones were either completely diploid (A,D) and had lower fluconazole MICs, or were aneuploid and had higher MICs (B,C). Clone D9-3.3-D (B) contains i(5L) and is trisomic for Chrs 3, 4, and 7, while clone D11-3.3-A (C) contains multiple copies of i(5L) and is trisomic for Chrs 3 and 7.

CGH of D9 strains was consistent with the CHEF karytoype gel analysis: no i(5L) or other aneuploidies were detected in clone D9-3.3-A, while i(5L) was detected in clone D9-3.3-D (Figure 4A and 4B). In addition, CGH revealed that Chr3, Chr4 and Chr7 were trisomic in clone D9-3.3-D. Thus, clone D9-3.3-D included multiple aneuploidies that were not evident on the CHEF karyotype gels and its MIC (∼3.0 µg/ml), was higher than that of clone D9-3.3-A (∼1.5 µg/ml), which had no obvious aneuploidies. This is consistent with the idea that aneuploidies in D9-3.3-D (including, but not limited to i(5L)) confer increased drug resistance. Importantly, the additional whole chromosome trisomies did not impair fitness under either the selective or non-selective growth conditions (Figure 3C) and were observed at generation 330 (discussed below).

Clones from population D11-3.3 (Figure 4C and 4D) were generally similar to the clones from population D9-3.3. D11-3.3-A was trisomic for Chr3 and Chr7 (but not for Chr4) and carried i(5L). Furthermore, as in D9-3.3 clones, the MIC of strains with multiple aneuploidies was slightly higher (∼2.0 µg/ml) than the MIC of strains lacking them (∼0.5 µg/ml). For D12-3.3, all single colony clones contained i(5L), and thus we could not compare their growth to sister clones lacking i(5L). Importantly, the degree to which D9-3.3, D11-3.3 and D12-3.3 had increased fitness in the presence and absence of drug correlated positively with the proportion of the population that contained aneuploidies (Figure 3C and Table 1).

Aneuploidies detected by CGH in evolved populations and their respective individual clones (Figures S6, S7), are summarized in Table 2 and revealed several interesting findings: First, the accumulation of multiple aneuploidies correlated with the highest MIC. Both D9 and D11 accumulated more aneuploidies than D12 over the evolution experiment and also reached a higher final MIC (96 µg/ml) than D12 (8 µg/ml). This is consistent with the idea that aneuploidy can promote evolvability under strong selection [22],[29],[35].

**Table 2:** *C. albicans* strains used (ploidy characterized by CGH).

Stain Name	Strain Number	Relevant Ploidy
SC5314	YJB2348	Diploid
T118	YJB9613	Diploid
T118-Eno1::*NAT*	YJB10821	Diploid
N1-330	YJB10653	Diploid
N2-330	YJB10654	Diploid
N3-330	YJB10655	Diploid
N4-330	YJB10656	Diploid
N5-330	YJB10657	Diploid
N6-330	YJB10658	Diploid
N1-330-A	YJB10881	Diploid
N2-330-A	YJB10883	Diploid
N3-330-A	YJB10885	Diploid
N4-330-A	YJB8741	Diploid
N5-330-A	YJB10888	Diploid
D8-330	YJB8734	Diploid
D9-3.3-A	YJB10952	Diploid
D9-3.3-D	YJB10955	i(5L),Trisomy Chr3, Chr4, Chr7
D9-165	YJB8735	i(5L), Trisomy Chr7
D9-260-A	YJB10970	i(5L), additional 5L, Trisomy Chr6, Chr7
D9-260-B	YJB10971	i(5L), additional 5L, Trisomy Chr6, Chr7
D9-260-B_CHEF band only	YJB10971-CHEFband	2× Chr5L and 1× Chr3R (*orf19.5999*- right telomere)
D9-260-F	YJB10975	i(5L), Trisomy Chr7
D9-330	YJB8736	i(5L), Monosomy 5R, Trisomy Chr4, Chr6, Chr7, Segmental Trisomy Chr3
D11-3.3-A	YJB10981	i(5L), Trisomy Chr3, Chr7
D11-3.3-D	YJB10984	Diploid
D11-200	YJB10673	2× i(5L), Trisomy Chr7
D11-260	YJB10675	2× i(5L), Trisomy Chr7
D11-330	YJB8737	2× i(5L), Monosomy 5R, Trisomy Chr7, Segmental Trisomy and Segmental Tetraploidy Chr4
D12-165	YJB8738	attached Chr5-i(5L), Trisomy Chr7
D12-165+100 generations without Flu selection	YJB10283	Trisomy Chr7
D12-260	YJB8739	Trisomy Chr4, Chr7
D12-330	YJB8740	Trisomy Chr4

Second, whenever i(5L) was present, Chr7 trisomy was also evident, suggesting that extra copies of Chr7 may enhance the fitness of strains carrying i(5L). However, loss of i(5L), detected only in the D12 series, was not accompanied by coincident loss of Chr7 (described below). Thus, i(5L) and Chr7 do not co-segregate during all changes in chromosome content.

Third, we observed dynamic appearance and subsequent disappearance of different clones/aneuploidies: in D9-3.3 the aneuploid genotype (i(5L) + trisomy of Chrs 3, 4 & 7) was present in ∼15% of the population; in D9-165 trisomy of Chr3 & Chr4 was not evident; in D9-230 and D9-300, a new chromosome band (Figure 2D arrow, discussed below) appeared and then disappeared; and, in D9-330, in addition to i(5L), Chr5R was monosomic, Chrs4, 6 and 7 were trisomic and Chr3 had a segmental trisomy. Nonetheless, whole chromosome and segmental aneuploidies detected in single clones at generation 3.3 were highly predictive of the aneuploidies detected in the D9 and D11 populations at generation 330. For example, the aneuploid chromosomes of clone D9-3.3-D (i(5L), Chr3, Chr4, & Chr7) were present in population D9-330, except that the Chr3 trisomy became a segmental trisomy. This suggests that, once it becomes aneuploid, the *C. albicans* genome is dynamic and continues to change. Yet, under fluconazole there was strong selective pressure for a similar repertoire of aneuploidies. Finally, no aneuploidy was detected in the progenitor strain (T118), in the endpoints of each N1 population (N1-330 to N6-330), nor in single colonies from within each endpoint population (N1-330-A to N6-330-A) (Figures S4, S5). Thus, during extended growth in vitro, in the absence of selective pressure, the karyotype did not change.

### Transient appearance of a supernumerary chromosome (SNC) is associated with decreased fitness yet higher MIC relative to the progenitor

**Figure 5:**
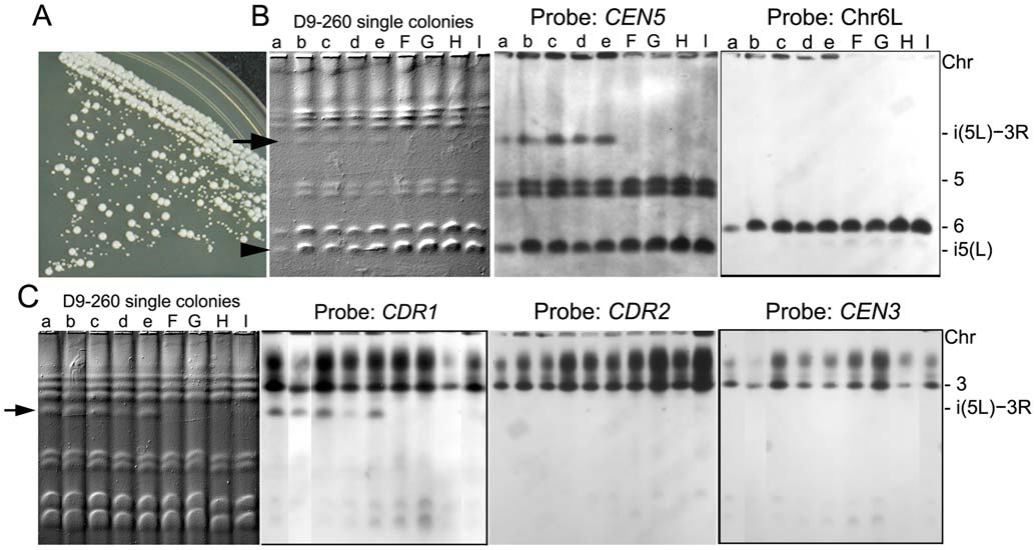
Southern blot analysis of the ∼1.5 Mb SNC. (A) Population D9-260 exhibited colony size variability on YPD plates. (B,C) CHEF/Southern analysis of single colonies detected a novel ∼1.5 Mb SNC in small (lanes a–e) but not in large (lanes f–i) colonies. The ∼1.5 Mb SNC hybridized to *CEN5* and Chr3R probe *CDR1* (arrows), but not to probes from probes further to the left on Chr3 (*CDR2* and *CEN3*) or from Chr6. Both small and large colonies maintained independent i(5L) (arrowhead).

**Figure 6:**
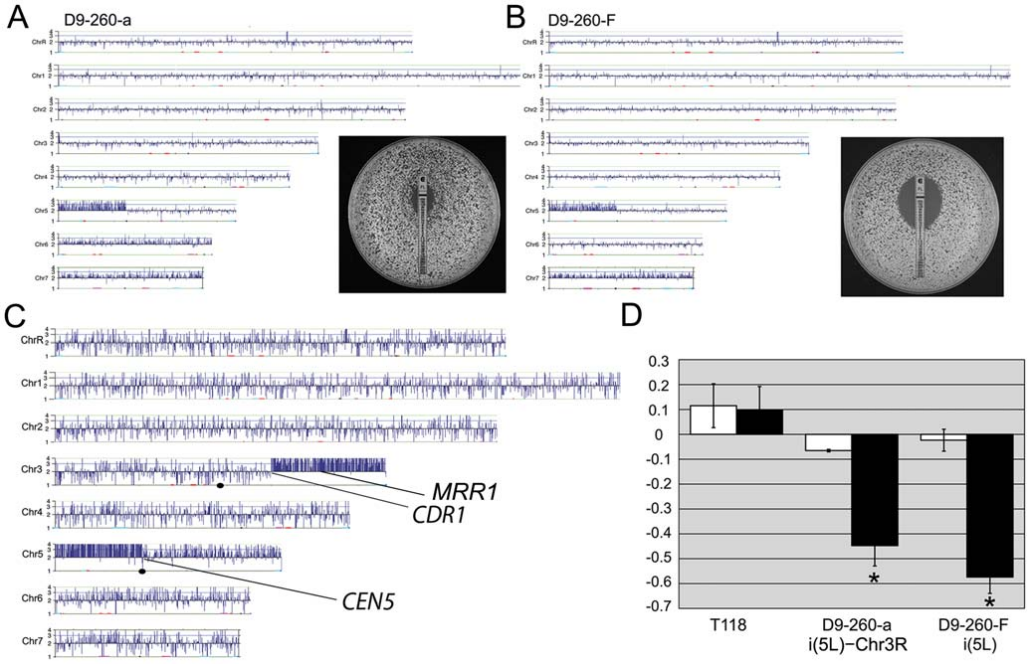
Supernumerary chromosome i(5L)-3R increases resistance to fluconazole. (A) CGH analysis of small colony D9-260-a detected ∼4 additional copies of Chr5L and trisomy of Chr6 and Chr7, while (B) CGH of a large colony D9-260-F identified ∼2 additional copies of Chr5L (i(5L)) and Chr7 trisomy. E-test strip assays indicated that the smaller colony MIC (∼64 µg/ml) (A, insert) was higher than the larger colony MIC (∼8 µg/ml) (B, insert). (C) CGH analysis of the isolated ∼1.5 Mb SNC DNA from D9-260-b. Genes from Chr5L (breakpoint at *CEN5*) and Chr3R (breakpoint just to the left of *CDR1*) are highly overrepresented on the ∼1.5 Mb SNC. (D) Fitness assays, of D9-260 clones are consistent with slow growth of both the small (D9-260-a) and larger (D9-260-F) colony clones relative to the progenitor, suggesting that the population has accumulated a large number of mutations. Nonetheless, small colony clones containing the ∼1.5 Mb SNC were slightly more fit in the presence of fluconazole (black bars, ∼128 µg/ml, twice the MIC of the experimental population D9-260) than clones that did not carry the SNC. Fitness of the aneuploid strains was not reduced significantly in the absence of fluconazole (white bars). Asterisks indicate fitness levels that are significantly different from the progenitor strain.

A new band with apparent size of ∼1.5 Mb appeared in the later D9 populations (D9-230, D9-260 and D9-300, Figure 2D, upper arrow) while i(5L) remained detectable in these populations. Clones from D9-260 grew with two distinct colony size phenotypes (Figure 5A): small colonies that grew slowly (clones D9-260-a to -e) and colonies with a wild-type size and growth rate (clones D9-260-F to -I). CHEF gel analysis indicated that the small colonies contained the ∼1.5 Mb SNC as well as i(5L) while the larger colonies retained i(5L) only (Figure 5B). CGH analysis of small and large colonies indicated that both types of colonies contained 2–4 additional copies of Chr5L and were trisomic for Chr7 (Figure 6A and 6B). In addition, small colony D9-260-a was trisomic for Chr6. To ask if the ∼1.5 Mb SNC included either Chr5R, Ch6 and/or Ch7 DNA fused to Chr5L DNA, we probed Southern blots of the CHEF with probes from Chr6 (two from 6L and one from 6R), *CEN7*, the right arm of Chr5, and *MTL*α on Chr5L (probes are listed in Table S1). None of these probes hybridized to the ∼1.5 Mb SNC (Figure 5B and data not shown). This suggests that neither Chr5R, nor Chr6, nor Chr7 are fused to Chr5L in the novel SNC.

We then isolated the ∼1.5 Mb SNC from the CHEF gel, labeled it with Cy3 and analyzed the contents of this single chromosome by CGH [21]. As expected, the band included DNA from all of Chr5L including *CEN5*. Strikingly, it also included a segment of Chr3R beginning just to the left of *DYN1* (*orf19.5999*) (Figure 6C). While this type of analysis cannot directly determine copy number (because one chromosome is hybridized relative to a whole genome), the signal from Chr5L was approximately twice as high as the signal from the Chr3R segment. This is consistent with the size of the fragment and implies that the ∼1.5 Mb SNC is composed of two copies of Chr5L (most likely organized as an isochromosome, ∼945 kb) attached to a (∼600 kb) segment from Chr3R (5L-CEN5-5L::3(*orf19.5999*→tel)). We refer to the ∼1.5 Mb SNC as i(5L)-3R.

The detection of a Chr3R segment in the isolated ∼1.5 Mb SNC was surprising since no obvious increase in the number of Chr3R copies was evident in the whole genome CGH of small colony D9-260 clones (Figure 6A and Figure S6B). Southern analysis of whole chromosome CHEF gels confirmed that *CDR1*, a gene just to the right of *DYN1* and within the Chr3R segment, is present on the ∼1.5 Mb SNC as well as on intact Chr3. and *CDR2*, a gene just to the left of *DYN1*, is not (Figure 5C). A *CEN3* probe, located farther to the left of the Chr3R segment breakpoint, is also absent from the ∼1.5Mb SNC (Figure 5C). Consistent with the fusion of Chr3R to an i(5L), the ∼1.5 Mb SNC did not include an *Sfi*1 restriction site (Figure S8A). Also, since both the independent i(5L) and the i(5L)-3R SNC in these clones carry only the *MTLa* homolog (Figure S8B), the event that gave rise to the ∼1.5 Mb SNC likely involved a non-reciprocal recombination event in which one Chr3R segment was copied onto the end of the i(5L) that was already present in the population.

The stability of the i(5L)-3R SNC was highly variable, with some small colony clones giving rise to all small colonies and some giving rise to small and large colonies. CHEF gel analysis indicates that all of these large colony derivatives had lost the ∼1.5 Mb SNC (Figure S8C). In fact, loss of the SNC occurred in isolate D9-260-a during subsequent propagation for CHEF analysis (∼20 generations in YPD) (Figure S8A, single colony lane “a”). These observations prompted analysis of fitness and chromosome stability in clones with and without the SNC. The slow growth of clones containing the ∼1.5 Mb SNC is consistent with the idea that this SNC caused a burden on cell growth. Indeed, it caused a growth disadvantage in the absence and in the presence of fluconazole relative to the progenitor strain (Figure 6D). The MIC of this subpopulation (48–64 µg/ml) accounts for the MIC of the original D9-260 population (64 µg/ml). Despite the very slow growth of the small D9-260 clones in the presence of drug, the large D9-260 clones grew even less well in fluconazole and had a much lower MIC (8 µg/ml). This implies that the SNC is a beneficial yet costly mutation (Figure 6D). Consistent with the observation that the i(5L)-3R SNC in clone D9-260-a was unstable during culture, a double ellipse of growth formed in the e-strip assay (Figure 6A, insert) one at 64 µg/ml and the other at 8 µg/ml. We propose this is due to loss of the SNC during growth of the colonies on the plate.

### Instability of att-i(5L)

**Figure 7:**
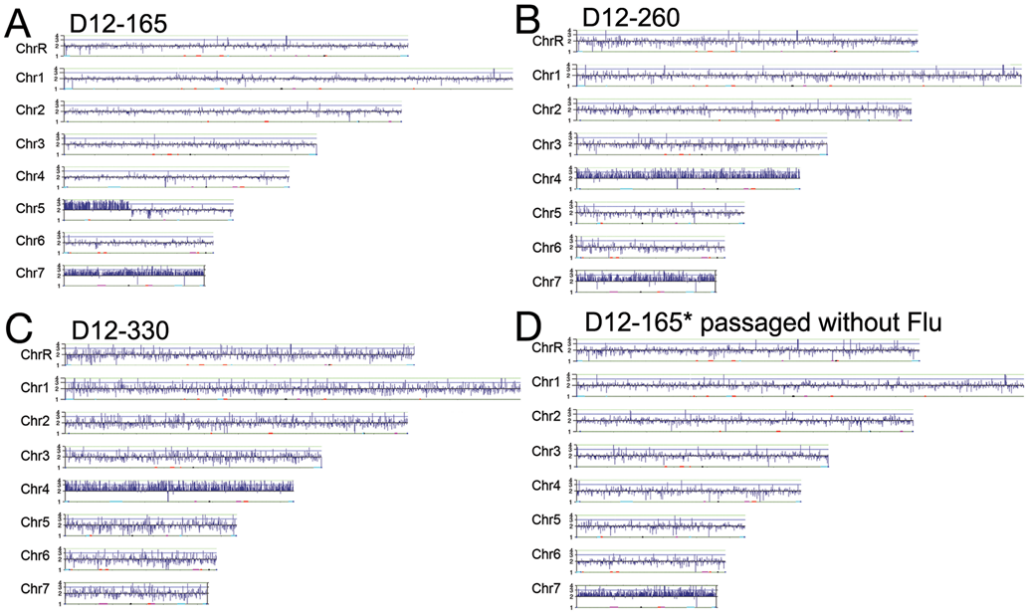
Changes in aneuploid chromosomes are common during adaptation to fluconazole. Population D12 underwent drastic changes in aneuploid chromosome number, which indicates that clonal interference was high, and coincides with a fluctuation in fluconazole MIC. (A) D12-165 has att-i(5L) and is trisomic for Chr7, (B) D12-260 lost the att-i(5L) and gained Chr4 trisomy, and (C) D12-330 lost the Chr7 trisomy, but maintained the Chr4 trisomy. (D) Population D12-165 was passaged for an additional ∼100 generations in the absence of fluconazole and it lost the att-i(5L) aneuploidy, but maintained the Chr7 trisomy.

**Figure 8:**
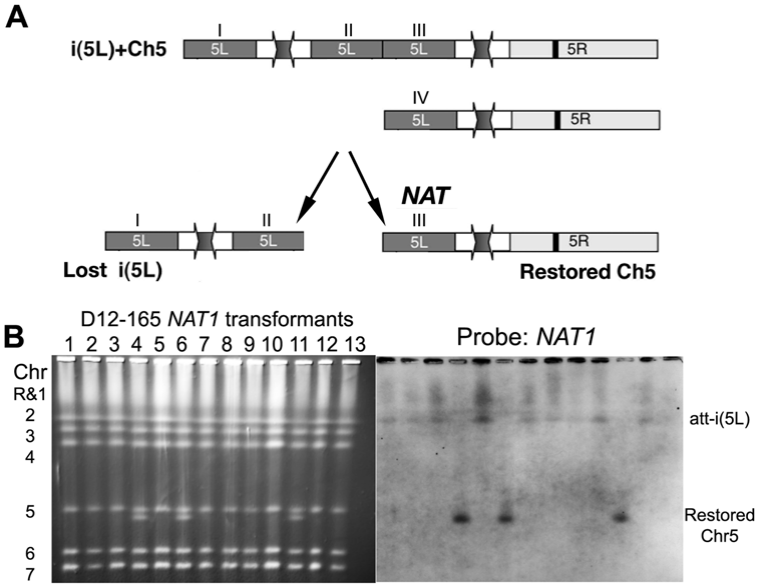
Transformation of att-i5L results in loss of i(5L) and retention of the intact Chr5 portion. (A) Strain D12-165 contains an att-i(5L) chromosome with two *CEN5* structures (top) and one normal Chr5 homolog (bottom). Transformation was used to disrupt *orf19.1963* on Chr5L with the *NAT1* marker. There are 4 existing *orf19.1963*, three on the att-i(5L) (I, II, III) and one on the full-length Chr5 homolog (IV). (B) CHEF/Southern blot analysis of 13 *NAT1* positive transformants indicates that in all but 3 cases the *NAT1* insertion occurred on the att-i(5L) at any locus I-III. In the remaining 3 transformants, *NAT1* insertion occurred at locus III and coincided with detachment of the i(5L) and reformation of the independent Chr5 homolog. This same phenomenon was observed during the original D12 evolution experiment.

In D12, the att-i(5L) was maintained through 230 doublings and then was quickly lost. CGH analysis of these strains detected the i(5L) DNA as well as trisomy of Chr7 (Figure 7A). No other aneuploidies were evident in the strain. Thus, an extra copy of Chr7 accompanied the acquisition of i(5L) in all three of the cultures derived from T118 that reached higher MICs (D9, D11 and D12). A drop in MIC accompanied the loss of the att-i(5L), and the intact *MTL*α Chr5 homolog, whose mobility was altered upon att-i(5L) formation, was restored (Figure 8A). A similar loss of att-i(5L) and restoration of the original Chr5 homolog was seen in transformants derived from D12-165 (Figure 8B). Importantly, the drop in MIC upon loss of att-i(5L) and retention of Chr7 trisomy (Figure 7B) suggests that the major contribution to fluconazole resistance came from genes on i(5L), rather than from genes on Chr7. Consistent with this, propagation of D12-165 (att-i(5L) and Chr7 trisomy) in the absence of drug for over 100 doublings (or transformation with *NAT1* which sometimes led to i(5L) loss) resulted in a drop in MIC, loss of the att-i(5L) from the population and retention of Chr7 trisomy (Figure 7D).

In the original study, continued evolution of D12-260 for another 70 doublings in the presence of fluconazole (D12-330) was accompanied by loss of Chr7 trisomy, while Chr4 trisomy remained in the culture (Figure 7C). Loss of the i(5L) and Chr7 trisomy resulted in a drop in MIC from 64 µg/ml to 4 µg/ml. This residual Flu^R^ (compared to the progenitor) could be due to the extra copy of Chr4 or to other mutations accumulated in the strain during propagation in the presence of the drug.

## Discussion

In this study we analyzed changes in genome organization and copy number that are now recognized as important contributors to antifungal drug resistance. *In vitro* analysis of the evolutionary process in strains with an identical ancestor allowed us to monitor the events that arise and change over time in parallel populations. Analysis of multiple clones at each generation allowed us to follow specific mutations that occurred at each generation and determine the benefit of those mutations based on their fixation within the population. Importantly, results from this experimental evolution study mirror the analysis of clinical Flu^R^ strains: in both cases ∼50% of the cultures (3/6 in this study) exhibited aneuploidy associated with the acquisition of Flu^R^ and the presence of i(5L) correlated with the highest fluconazole MICs. Furthermore, loss of i(5L) correlated with a drop in fluconazole MIC levels [11],[20]. The most surprising result is the early appearance of cells carrying multiple aneuploidies including, but not limited to i(5L). Yet different types of i(5L) were acquired in 3 populations while i(5L) did not appear early in the other three drug-treated populations. Importantly, neither i(5L), nor any other aneuploidy, was detectable in any of the populations that were not exposed to drug. This suggests that under fluconazole stress, either fluconazole is a mutagen or *C. albicans* cells enter a hypermutable state when exposed to fluconazole [34]. Based on the standard exponential growth model of cell division, cells carrying i(5L) were inadvertently enriched during storage and resuscitation of the original isolates (see Materials and Methods). Nonetheless, the presence of i(5L) in these populations, irrespective of their prevalence, indicates that they were present; their fitness advantage indicates that they became increasingly prevalent during propagation in drug.

### How does aneuploidy arise so rapidly?

i(5L) was detectable by CHEF in all three populations after one 24-hour passage in fluconazole. During this time the population had undergone an average of 3.3 doublings in the presence of fluconazole. Based on the exponential growth rate model (Materials and Methods) we assume that i(5L) arose early after exposure to fluconazole although we cannot rule out the possibility that a very small number of i(5L) cells existed in the progenitor culture. If i(5L) appeared early after exposure to fluconazole, it would support the model that stress-induced mutagenesis may operate in *C. albicans* [34]. Consistent with this, stress response signaling through Hsp90 and/or calcineurin is required for fluconazole tolerance [36]–[38]. Alternatively, fluconazole may function (directly or indirectly) as a mutagen: it has been reported to be clastogenic in mouse bone marrow cells [39] and causes significantly increased levels of loss of heterozygosity in *C. albicans* (A. Forche and JB, in preparation).

The frequency with which *C. albicans* undergoes major chromosome rearrangements in fluconazole is apparently higher than the frequency of such events in *S. cerevisiae* haploid or diploid cells [40]. One explanation offered for the plasticity of the *C. albicans* genome is that it is not subject to the constraints imposed by meiotic segregation and thus it can retain detectable genome rearrangements that would be lost in organisms that undergo meiosis. Alternatively, *C. albicans* may lack some cell cycle checkpoints that prevent aneuploidy in other organisms, or it may have an active mechanism to induce concerted chromosome changes like those seen during the parasexual cycle [41]. Whatever the mechanism, it is becoming increasingly clear that aneuploidies are more prevalent than originally thought. For example, ∼8% of the original *S. cerevisiae* deletion collection contained aneuploidies, most of them segmental [42]. Furthermore, in the presence of strong selective pressure, *S. cerevisiae myo2*Δ cells with defects in cytokinesis evolved alternative mechanisms of cell division via the acquisition of aneuploidies for specific sets of chromosomes [22]. Similarly, whole chromosome trisomy and higher copy aneuploidies were detected more frequently in diploid than haploid *S. cerevisiae* cells evolved under nutritional stresses [29]. Thus, while the frequency of aneuploidy in *C. albicans* (a diploid) is high, *S. cerevisiae* diploids also undergo similar types of events when placed under selection pressure. The lack of detectable aneuploidy in untreated cells (Figures S4, S5) and detection of high rates of aneuploidy in drug treated cells ([11] and this study), as well as in strains isolated from patients [20] or from animals [43] suggests that *C. albicans* is exposed to constant selective pressure when it is propagated in mammalian hosts. We suggest that this selective pressure may be even stronger when *C. albicans* is exposed to antifungal drugs within the mammalian host.

### Variations on Chr5L geometry and copy number

Chr5L appeared in the evolved strains as an independent chromosome of ∼945 kb throughout the experiment in two of the cultures (D9 and D11), as an i(5L) attached to a full length Chr5 (D12), and as an i(5L) attached to a segment of Chr3R in later D9 populations. Chr5L copy number also varied in these strains, irrespective of geometry. For example, multiple copies of independent i(5L) were present in D11 cells (Figure S7); CGH estimates approximately 6 copies of Chr5L. Similarly, D9-260 clones contain Chr5L sequences on 4 different chromosomes with 3 different geometries: intact Chr5 (2 copies), i(5L), and the i(5L)-3R SNC.

In D12, att-i(5L), which includes centromere-to-centromere and telomere-to-telomere orientations of Chr5L, was acquired early and was maintained for over 200 doublings. This structure is potentially dicentric, however micrococcal nuclease digestion studies suggest that one of the three copies of *CEN5* in the strain is not packaged into centromeric heterochromatin (Carrie Ketel, unpublished data) and thus is not functional. We presume this is the *CEN5* DNA on the i(5L) portion of att-i(5L) since, once detached (in D12-260), this DNA is not stably maintained (Figure 8). In addition, in the D9 culture the i(5L)-3R SNC appeared in a subpopulation of cells and was maintained in the population for 70 doublings (D9-230 through D9-300).

Chr5 is naturally heterozygous, because it carries the mating type-like locus *MTL*. An *MTL*α probe hybridized to att-i(5L) while an *MTLa* probe did not, implying that att-i(5L) includes three copies of the Chr5L homolog that carries *MTL*α. In contrast, the i(5L) in both D9 and D11 isolates as well as the i(5L)-3R SNC hybridized to *MTLa* and not to *MTL*α. Thus, independent i(5L) as well as attached i(5L) derivatives can form from either Chr5 homolog, yet all copies of i(5L) within a single cell contain the same *MTL* allele. Importantly, because each isochromosome is homozygous for one *MTL* allele, the mechanism of isochromosome formation likely involves gene conversion or break-induced replication rather than a reciprocal recombination event between two copies of Chr5. Furthermore, in contrast to a previous study in which *MTL* homozygosity was associated with Flu^R^ [44], here, *MTL* heterozygosity was maintained throughout all three evolution experiments. Rather, extra copies of Chr5L (and thus of *MTL*) correlated with increased Flu^R^, most likely due to the additional copies of *ERG11* and *TAC1* that contribute independently and additively to Flu^R^ [20].

### Other aneuploidies in Flu^R^ strains

In addition to Chr5L aneuploidy, increased gene copy numbers of chromosomes 3, 4, 6, and 7 were found. Multiple genes known to be important for Flu^R^ are found on these chromosomes: The segmental trisomy of Chr3 in strain D9-330 (Figure S6) and Chr3R segment attached to i(5L) on the i(5L)-3R, includes *CDR1*, which encode the major ABC-transporter important for Flu^R^ [45],[46], as well as *MRR1*, which encodes a transcription factor that up-regulates MDR efflux pumps [18]. *MDR1* is found on Chr6. Increased gene copy number of Chr3 and/or Chr6 is likely the reason that *MDR1* is expressed at increased levels in this strain [28]. Additionally, Chr4 copy number was increased in isolates from all three series: D9-3.3-D, D9-330, D12-260 and D12-330 exhibited Chr4 trisomy, and a segmental trisomy of Chr4 in D11-260 was amplified to even higher levels in D11-330. *NCP1*, which encodes NADPH-cytochrome P450 reductase and is a co-factor of Erg11p (the target of fluconazole) in sterol 14 alpha-demethylation in ergosterol biosynthesis, is on Chr4 within the most amplified region in D11-300 (>5 gene copies). We propose that extra copies of Chr4 may provide increased levels of Flu^R^ because of the increased levels of *NCP1*. Additional genes on Chr4 that might affect Flu^R^ included *ERG8*, *ERG27* and *ERG251*, which are all involved in ergosterol biosynthesis. *ERG251* is upregulated in response to at least one azole drug, ketoconazole [47], but it is not known if increased numbers of these genes contribute to drug resistance. Thus, several of the aneuploid chromosomes that became prevalent in the drug treated populations carry genes that have already been shown to be important for increased resistance to fluconazole.

### Aneuploidy in *C. albicans*, as in cancer cells, does not have a high fitness cost

Malignant cancer cells exhibit unrestricted growth and high levels of aneuploidy and polyploidy. Yet most aneuploid *S. cerevisiae* strains exhibit growth defects under non-selective conditions [48]. This raises an important conundrum: How is it that aneuploidy in cancer cells does not restrain growth, yet aneuploidy in model organisms does? In stark contrast, aneuploidy in *C. albicans*, like aneuploidy in cancer cells, does not appear to have a high fitness cost. All aneuploidies reported here all were detectable after passage for ≥70 generations in non-selective medium (YPD). Other Flu^R^ strains also have been propagated extensively under non-selective conditions without loss of i(5L). *C. albicans* aneuploidies are usually increased copies of whole chromosomes whereas *S. cerevisiae* aneuploidies that arise in haploid cells under selection are primarily shorter segmental aneuploidies [21],[29],[32]. As an obligate diploid, *C. albicans* may be better able than haploids to tolerate the acquisition of an extra chromosome (50% vs 100% increase in copy number, respectively). Accordingly, *S. cerevisiae* diploids exhibit higher rates of aneuploidy under strong selection [29]. An argument against this simple model is that mice carrying specific trisomies exhibit reduced fitness [49], however we cannot rule out the possibility that some aneuploidies or combinations of aneuploid chromosomes provide a selective advantage under stress conditions that were not tested in these experiments [50]. The concerted appearance of i(5L) and Chr7 trisomy are consistent with such an idea. We suggest that the acquisition of aneuploidy may be a general mechanism for the rapid evolution of genome change in response to severe stress conditions in *C. albicans*. At least in *C. albicans*, aneuploidy appears to arise frequently and is well tolerated, especially in cells responding to physiologically relevant concentrations of fluconazole. Thus, *C. albicans* and its response to fluconazole, may be an excellent model system for studying the molecular mechanisms that mediate genome changes that occur in mammalian cells in response to carcinogens.

## Materials and Methods

### Strain propagation and single-colony analysis

All populations were stored during the initial evolution experiment by freezing in growth medium plus 15% glycerol. These populations were revived by streaking to YPD, transferred between labs on filter paper, revived on YPD, stored in YPD+15% glycerol as a mixed population and then analyzed by CHEF and CGH, a process that involved ∼50–70 generations of growth on rich medium that did not contain fluconazole. For single colony analysis, clones were derived from a population plated on YPD directly from −80°C storage. After two days at 30°C single colonies were picked into 5 mL YPD liquid and grown up overnight also at 30°C. Liquid cultures were used to make CHEF agarose plugs (1% low melt agarose), gDNA for CGH or PCR analysis, and were again saved in 15% glycerol at −80°C. When variation in colony size morphology was observed after plating on YPD, multiple single colonies of each type were analyzed.

#### Measurement of Relative Fitness

Fitness of isolates from the experimental populations was determined by placing each isolate in direct competition with the progenitor, genetically marked with resistance to nourseothricin (*NAT1*). The progenitor was marked by C-terminally tagging *ENO1* with GFP using a *NAT1* selectable marker. The tagging construct was amplified by PCR with primers (Table S1) oLC597 and oLC596 from plasmid pMG2021 [20] and transformed into *C. albicans* following standard protocols. Transformants were tested for proper integration of the marker by PCR amplification with primers oLC598 and oLC600 as well as with primers oLC599 and oLC601. There was no significant difference in fitness between the marked and unmarked progenitor. Competition experiments were conducted in triplicate both in the presence and in the absence of fluconazole, essentially as described [33]. All competitions were conducted in the same medium as used for the evolution experiment [6], RPMI 1640, at 30°C. For the competitions that were conducted in the presence of drug, the concentration of fluconazole used was twice the most recently measured MIC of the experimental population. The competing populations were first conditioned by growing each competitor from the frozen archive separately overnight in RPMI 1640 medium. Cell counts of the overnight cultures, performed with a hemocytometer, were used to prepare a competition mix containing approximately equal concentrations of the two competitors. One hundred microliters of the competition mix was used to inoculate 9.9 ml of fresh medium (with or without fluconazole), and the competitors were allowed to grow together for 24 hours. Initial and final densities of each culture were determined by colony counts from dilution plates on yeast extract peptone dextrose (YPD) medium. The initial and final densities of each competitor were determined by replica plating the dilution plates onto YPD+NAT; under these conditions, only the genetically marked progenitor is able to grow. Fitness was estimated as the difference in number of doublings of the two competitors (evolved population minus the genetically marked progenitor), standardized by the total number of doublings in the competition assay, as described [33].

### Comparative Genome Hybridization

CGH analysis was performed as described previously using arrays produced in house [25]. The same reference control strain, SC5314, was used for all arrays in this paper. Multiple control hybridizations of SC5314 versus itself were performed and a representative example is provided in Figure S2A. Genomic DNA was isolated from cells grown in YPD and was RNaseA treated. 3 µg of DNA was digested with *Hae*III for 3–4 hours, purified, and then labeled with Cy3-dUTP (strain of interest) or Cy5-dUTP (SC5314) (Pharmacia/Amersham) using the BioPrime Array CGH labeling Module (Invitrogen). Labeled DNA was purified on a Microcon 30 filter (Millipore), mixed in a 1∶1 ratio, and applied to the array for a 16-hour hybridization. Fluorescence intensity ratios (mean log2 values) were obtained and analyzed using GenePixPro 5.1 and GeneTraffic 3.1. A Matlab program was used to plot all log2 data as a function of chromosomal location [25]. The CGH array data are listed in GEO, the Gene Expression Omnibus, as series “CGH analysis of *Candida albicans* strains after in vitro evolution in the presence or absence of fluconazole” with accession number GSE16423.

### CHEF and *Sfi*I digestion

Preparation of cells for CHEF analysis was performed as described previously [25]. Briefly, cells are grown up overnight in liquid YPD and gently collected by centrifuge and washed several times with 50 mM EDTA. Cells are suspended in 1% low melt agarose and then treated with BME and with Proteinase K solutions. Whole chromosome separation was performed on a BioRad CHEF-DR III with the following program: 60- to 120-s switch, 4.5 V·cm−1, 120°angle, for 36 hours, followed by a 120- to 300-s switch, 6.0 V·cm−1, 120°angle, for 12 hours. *Sfi*I digestion of CHEF gel plugs was performed as described previously [11] and separation of these large chromosome fragments was done with the following program: 7- to 100-s switch, 4.5 V·cm−1, 120°angle, for 21 hours, followed by 80- to 400-s switch, 3.5 V·cm−1, 120° angle, for 21 hours. CHEF gels were stained with Ethidium bromide and transferred to Magnacharge membranes for Southern blot analysis.

### CHEF–Southern blot analysis

All Southern blots were performed with DIG labeled probes (Roche) as described previously [25]. Probes were constructed by PCR using DIG-11-dUTP nucleotides (according to the manufacturer's instructions) and primers that are listed in Table S1.

### CGH analysis of an isolated CHEF band

Isolation, labeling and hybridization of the ∼1.5 Mb SNC was performed based on the PFGE-array protocol developed by Maitreya Dunham (http://dunham.gs.washington.edu/protocols.shtml). A whole-chromosome CHEF gel was run as described above with 5 lanes of strain YJB10971. The gel was stained with EtBr and the band representing the novel ∼1.5 Mb SNC was excised from all 5 lanes. The gel pieces were incubated twice for 1 hour in 4 volumes water and then incubated in 1× *Hae*III buffer (Roche) for 1 hour. Fresh 1× *Hae*III buffer and 5 µl *Hae*III enzyme (10 units/µl, Roche) were added and incubated overnight at 37°C. DNA was eluted from the gel slice and precipitated with salt and isopropanol. DNA was quantitated using a Nanodrop spectrophotometer and 700 ng of DNA was labeled with Cy3 (as described above), mixed with 700 ng of Cy5-labeled gDNA from SC5314 (as described above), and hybridized to a whole genome microarray.

### Fluconazole E-test

Cells were grown to mid-log phase, washed in 0.85% NaCl, and diluted to an OD600 of 0.01. 200–250 µl of cells were plated on Casitone-agar plates using glass beads and allowed to dry for 15–30 minutes before applying a fluconazole E-test strip (0.016–256 µg/ml, AB Biodisk). Plates were incubated at 30°C for 48 hours and the MIC was determined as the first growth-inhibition ellipse.

### Exponential growth rate modeling and freeze-thaw analysis

Doubling times were determined by growing mixed populations as well progeny of colonies that contained or did not contain i(5L) in RPMI or RPMI with fluconazole at 0.5 µg/ml, 35°C or in YPD medium at 30°C and at 35°C. D9-3.3-D strains (carrying i(5L)) have doubling times of ∼138 min (+/−10 min) in 0.5 µg/ml fluconazole, and thus could have divided up to 10.4 times in 24 hours. In contrast, the progenitor strain (without i(5L)) had a doubling time of ∼126 min (+/−9 min) in 0.5 µg/ml fluconazole; these cells consistently divided twice and then stopped dividing. Similar results were seen when T118 progenitor cells were grown in fluconazole and analyzed by time-lapse microscopy. Modeling of the exponential growth phase in the presence of drug based on these data indicates that if i(5L) cells divided once every ∼2.3 hours (138 min), and the non-i(5L) cells divided only twice in the 24 hour period, and if ∼1×10∧5 cells/ml were used to start the 24 hour culture (a number that was determined by measuring cell number after preparing starter cultures using conditions identical to those used in the original experiment [6]. If we assume one i(5L) cell appeared within first 1–2 hours after culture dilution into RPMI + fluconazole, that cell could have reached ∼0.3% of the culture after 24 hours. Because cells were stored in medium containing fluconazole, cells would have remained under selective pressure when resuscitated if residual drug remained active in the stored cultures and was not diluted upon streaking of cells from the frozen stock vial. In this case, cells containing i(5L) could have reached 15–20% of the culture over the next 16 hours of growth, based on the assumptions about growth rates as detailed above.

We performed several assays to determine if D9-3.3-D cells (with i(5L)) have any growth advantage on YPD media. We mixed progenitor and D9-3.3-D cells in a 1∶1 mixture and a 1∶99 (i(5L) to progenitor) mixtures. The mixtures were plated on YPD before freezing, after one freeze-thaw, and after three freeze-thaw cycles. In none of these experiments did D9-3.3-D cells have a growth advantage relative to the progenitor.

## Supporting Information

Figure S1 Single colony karyotype analysis of parental strain T118 and D9 populations. CHEF gel analysis followed by hybridization of the Southern blot with a probe to *CEN5* revealed that all single colonies derived from T118 had the same karyotype including two, separable Chr5 homologs. CHEF/Southern analysis of single colonies from generations D9-3.3, D9-140, D9-200, and D9-260 identified i(5L) (arrowhead) in some or all of the clones analyzed. In D9-260, an additional ∼1.5 Mb SNC hybridized to *CEN5* (arrow).
(7.16 MB TIF)

Figure S2 Single colony karyotype analysis of D11 generations D11-3.3, D11-50, D11-140, D11-200, D11-260, and D11-330. CHEF gel analysis followed by Southern blot with a probe to *CEN5* shows that the percentage of clones within each population that carried i(5L) (arrowhead) varied from 50% to 100% during the evolution experiment (summarized in Table 1).
(10.04 MB TIF)

Figure S3 Single colony karyotype analysis of D12 generations D12-3.3, D12-50, D12-230, and D11-260. CHEF gel analysis followed by Southern blot with a probe to *CEN5* shows that the percentage of clones within each population that carried the att-i(5L) (arrowhead) varied from 20% to 100% during the evolution experiment (summarized in Table 1).
(7.80 MB TIF)

Figure S4 Comparative genome hybridization of (A) the ancestral strain T118, (B) the *NAT1*-marked clone of T118 used for all fitness competitions, (C) population N1-330, (D) population N2-330, (E) population N3-330, (F) population N4-330, (G) population N5-330, (H) population N6-330. No aneuploidy was detected in these populations.
(4.78 MB TIF)

Figure S5 Comparative genome hybridization of the reference control SC5314 versus SC5314 (A) and of single colonies from populations: (B) N1-330, (C) N2-330, (D) N3-330, (E) N4-330, and (F) N5-330. No aneuploidy was detected in these clones.
(3.66 MB TIF)

Figure S6 Comparative genome hybridization of D9. (A) D9-165 contains i(5L) and Chr7 trisomy, (B) in clone D9-260-b the i(5L) with additional Chr5L copies and Chr6 and Chr7 trisomies, while (C) D9-330 has i(5L), trisomy of Chr4, Chr6, and Chr7, Chr5 monosomy, and a small increase in copy number on the right arm of Chr3 (at the same breakpoint found on the i(5L)-3R chromosome, asterisk).
(5.42 MB TIF)

Figure S7 Comparative genome hybridization of aneuploid D11 populations. All 3 populations, (A) D11-200, (B) D11-260, and (C) D11-330 have the i(5L) and are trisomic for Chr7. These populations also have increased copy numbers of i(5L), ∼2 copies per cell, based on Log2 values (Average Log2 values of Chr5L from two arrays 1.04+/−0.01, compared to the Average Log2 value of trisomic Chr7 from these same arrays of 0.41+/−0.09). D11-260 has a segmental trisomy on Chr4 that either gets further amplified or becomes fixed in the population, such that by generation 330 the region is highly anueuploid: it includes three copies of the entire right arm of Chr4 and at *CEN4* there is a transition to more than four copies of part of the left arm of Chr4. This amplified region includes *NCP1*, which encodes NADPH-cytochrome P450 reductase and is a co-factor of Erg11p. Finally, in population D11-300, Chr5R is monosomic.
(5.51 MB TIF)

Figure S8 Further characterization of the i(5L)-3R SNC. (A) CHEF gel analysis of *Sfi*I digested chromosomal DNA followed by Southern hybridization to a *CEN5* probe identified three bands (diagramed in D): the centromere-containing 5M band from intact Chr5 (∼750 kb), the full-size i(5L) (∼945 kb), and the full-size SNC (∼1.5 Mb). Hybridization with a probe to the right arm of Chr5 only detects the ∼750 kb 5M band (right panel). (B) The i(5L)-3R SNC and the independent i(5L) in D9-260 colonies both hybridize to the *MTLa* allele, and not to the *MTL*α allele. The same *Sfi*I-digested CHEF/Southern from (A) was probed with *MTLa* (left panel) and with *MTL*α (right panel). Bands representing i(5L)-3R, independent i(5L), and the *Sfi*I-digested Chr5M are indicated. (C) The small colony phenotype of D9-260 is due to the presence of i(5L)-3R. Ethidium Bromide-stained CHEF gel of single colonies derived from either a small (sm) or large (LG) colony from the D9-260-b population. CHEF gel plugs were prepared from a small colony that gave rise to another small colony (sm→sm), a small colony that gave rise to a large colony (sm→LG), or a large colony that gave rise to another large colony (LG→LG). Only small colonies maintained the i(5L)-3R SNC, while large colonies derived from the small colonies had lost the chromosome. (D) Diagram of a full-length Chr5 homolog depicting the location of the CEN5 probe, the Chr5R probe and the sole *Sfi*I restriction site, the digested ∼750 kb fragment from Chr5, the i(5L) lacking any *Sfi*I sites and the ∼1.5 Mb SNC composed of 2 arms of Chr5L, one *CEN5*, and part of Chr3R, which also has no *Sfi*I sites.
(3.46 MB TIF)

Table S1 Probes and primers used in this study.
(0.13 MB DOCX)
