# Supplementary figures and images for: Acquisition of Aneuploidy Provides Increased Fitness during the Evolution of Antifungal Drug Resistance
Source: PLoS Genet. 2009 Oct 30;5(10):e1000705. doi: 10.1371/journal.pgen.1000705 (PMC2760147; doi:10.1371/journal.pgen.1000705)

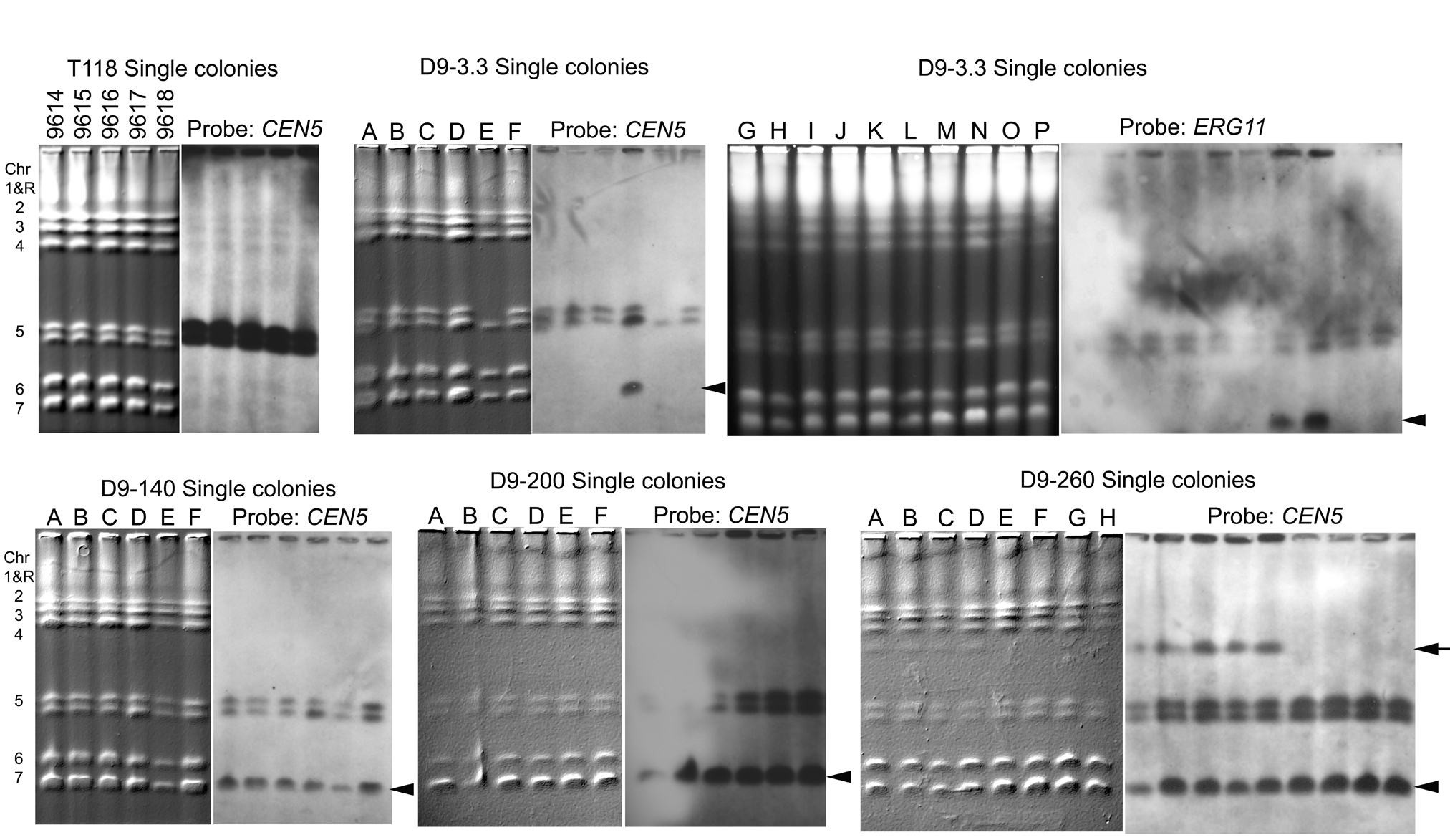

Supplement: Figure S1 — Single colony karyotype analysis of parental strain T118 and D9 populations. CHEF gel analysis followed by hybridization of the Southern blot with a probe to CEN5 revealed that all single colonies derived from T118 had the same karyotype including two, separable Chr5 homologs. CHEF/Southern analysis of single colonies from generations D9-3.3, D9-140, D9-200, and D9-260 identified i(5L) (arrowhead) in some or all of the clones analyzed. In D9-260, an additional ∼1.5 Mb SNC hybridized to CEN5 (arrow). (7.16 MB TIF) [file pgen.1000705.s001.tif]

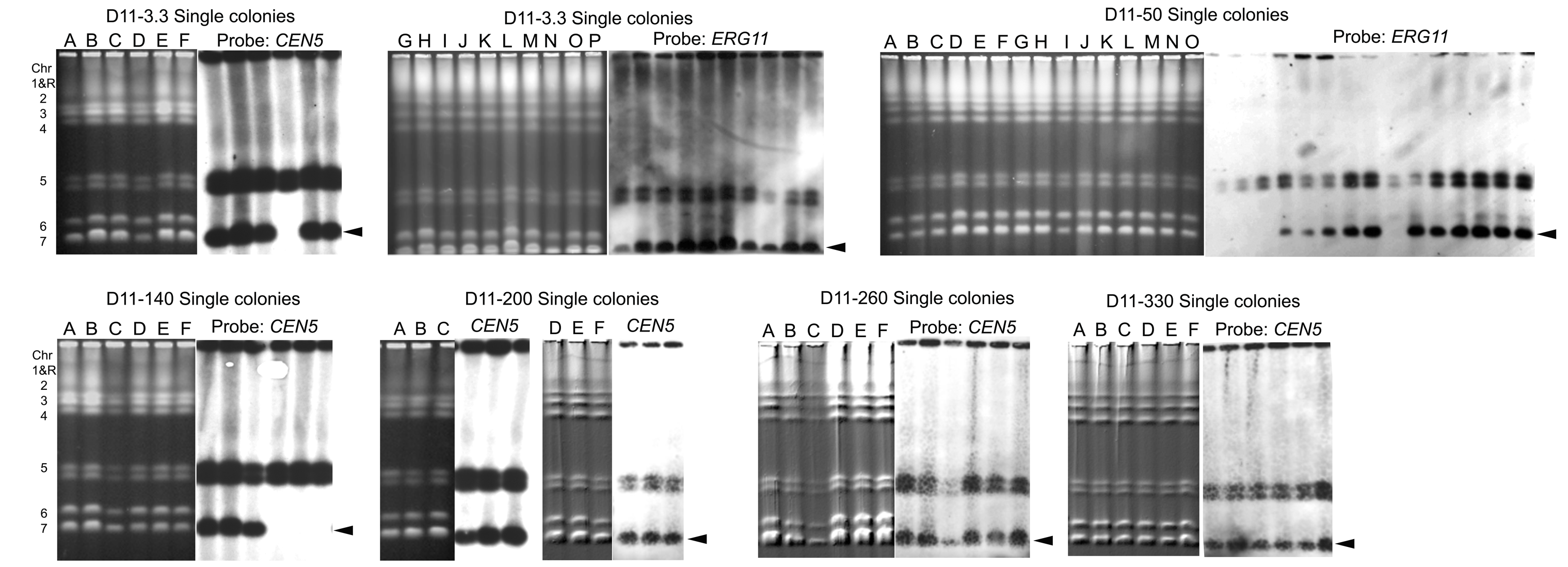

Supplement: Figure S2 — Single colony karyotype analysis of D11 generations D11-3.3, D11-50, D11-140, D11-200, D11-260, and D11-330. CHEF gel analysis followed by Southern blot with a probe to CEN5 shows that the percentage of clones within each population that carried i(5L) (arrowhead) varied from 50% to 100% during the evolution experiment (summarized in Table 1). (10.04 MB TIF) [file pgen.1000705.s002.tif]

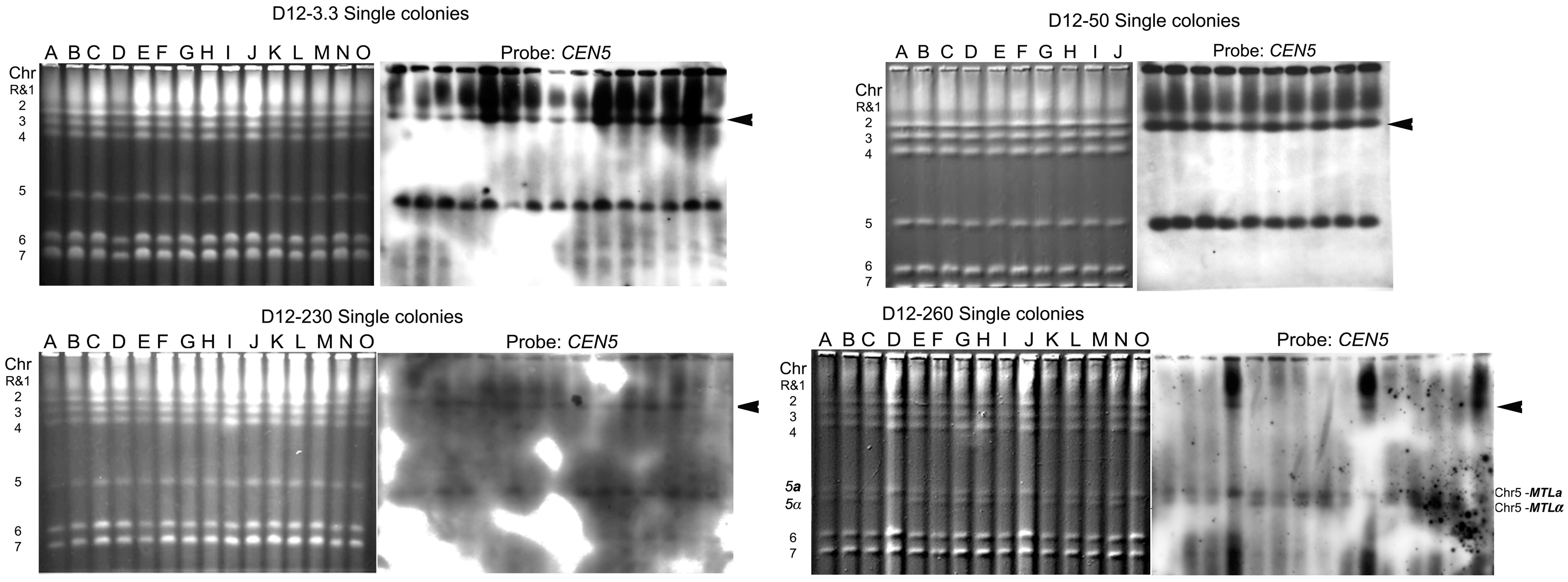

Supplement: Figure S3 — Single colony karyotype analysis of D12 generations D12-3.3, D12-50, D12-230, and D11-260. CHEF gel analysis followed by Southern blot with a probe to CEN5 shows that the percentage of clones within each population that carried the att-i(5L) (arrowhead) varied from 20% to 100% during the evolution experiment (summarized in Table 1). (7.80 MB TIF) [file pgen.1000705.s003.tif]

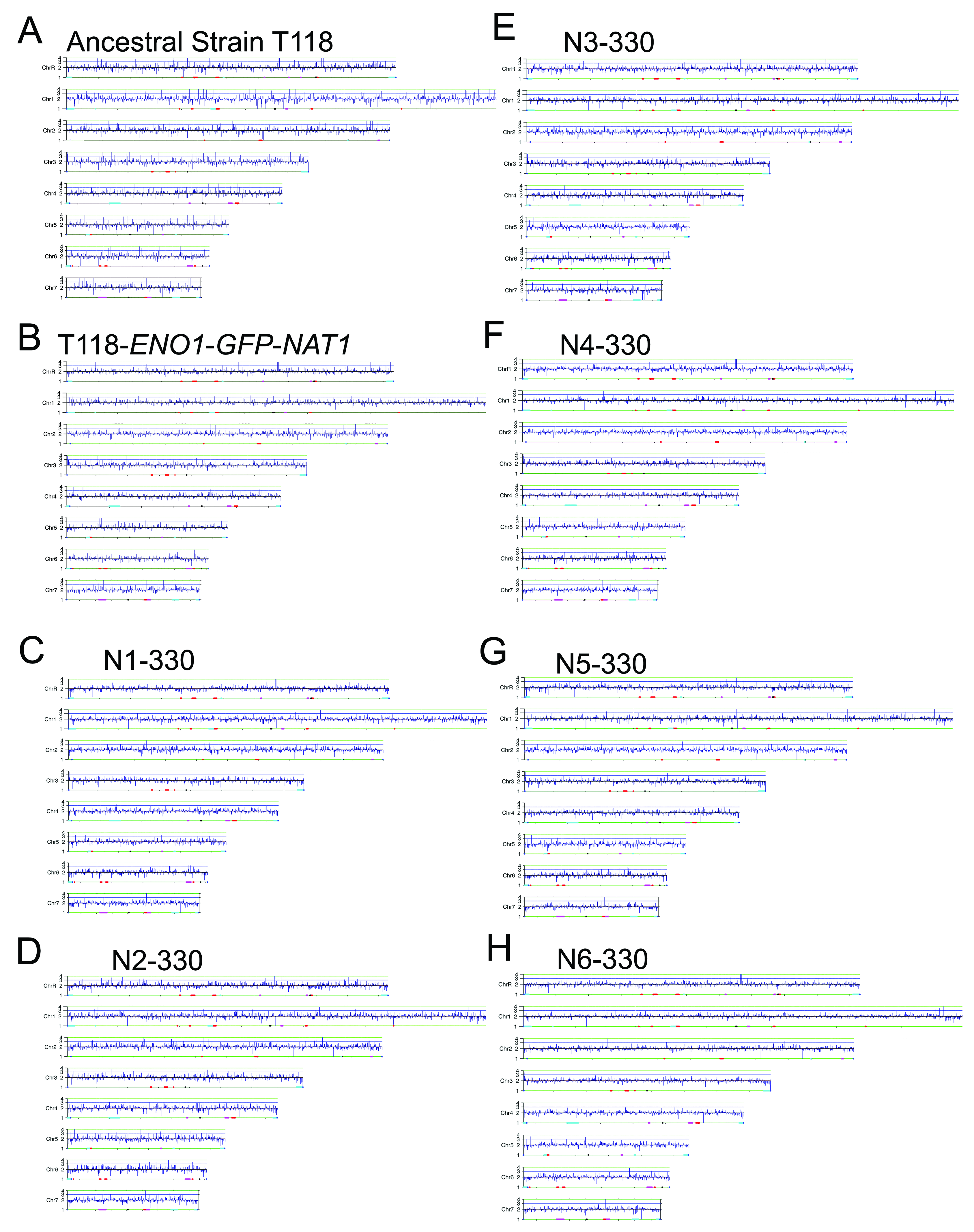

Supplement: Figure S4 — Comparative genome hybridization of (A) the ancestral strain T118, (B) the NAT1-marked clone of T118 used for all fitness competitions, (C) population N1-330, (D) population N2-330, (E) population N3-330, (F) population N4-330, (G) population N5-330, (H) population N6-330. No aneuploidy was detected in these populations. (4.78 MB TIF) [file pgen.1000705.s004.tif]

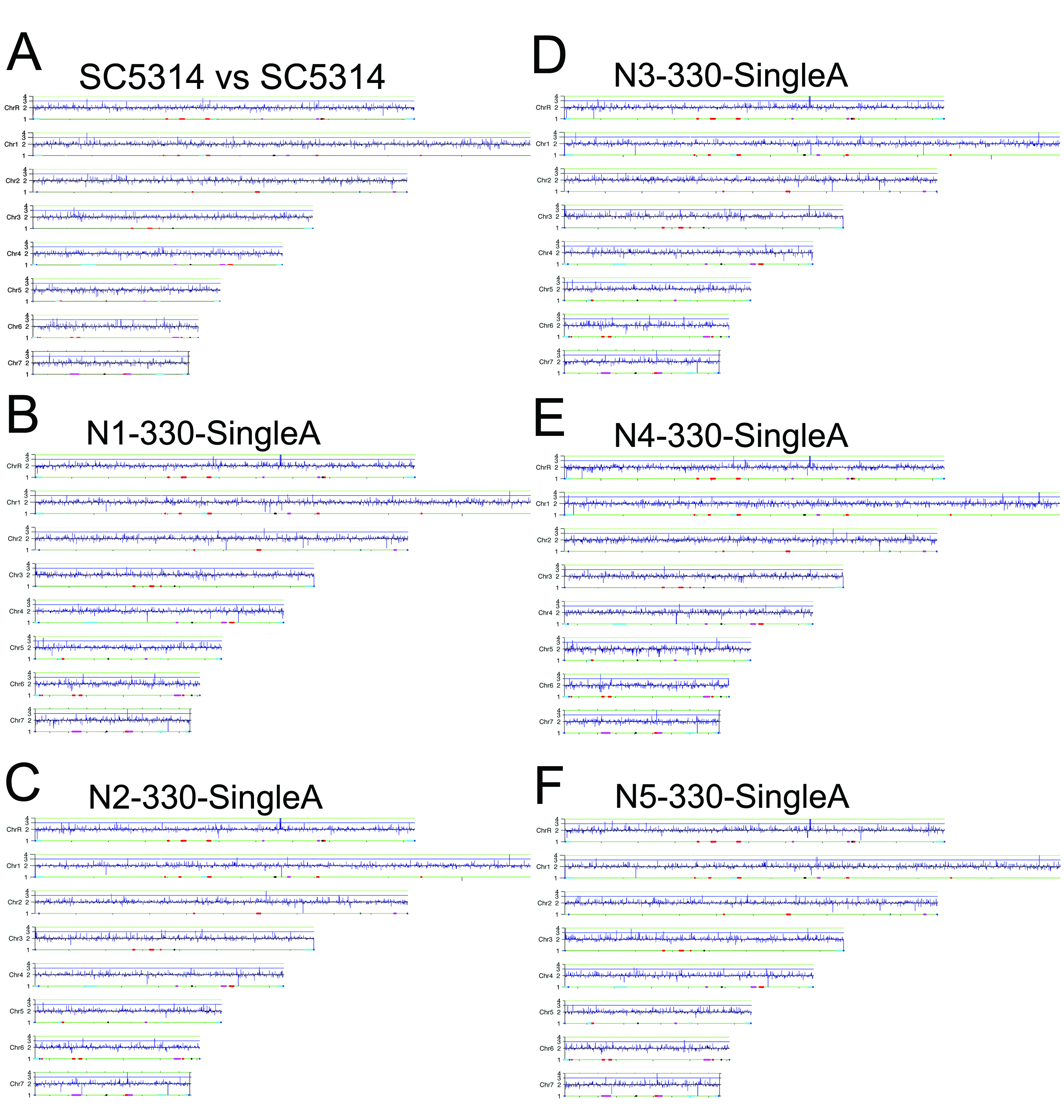

Supplement: Figure S5 — Comparative genome hybridization of the reference control SC5314 versus SC5314 (A) and of single colonies from populations: (B) N1-330, (C) N2-330, (D) N3-330, (E) N4-330, and (F) N5-330. No aneuploidy was detected in these clones. (3.66 MB TIF) [file pgen.1000705.s005.tif]

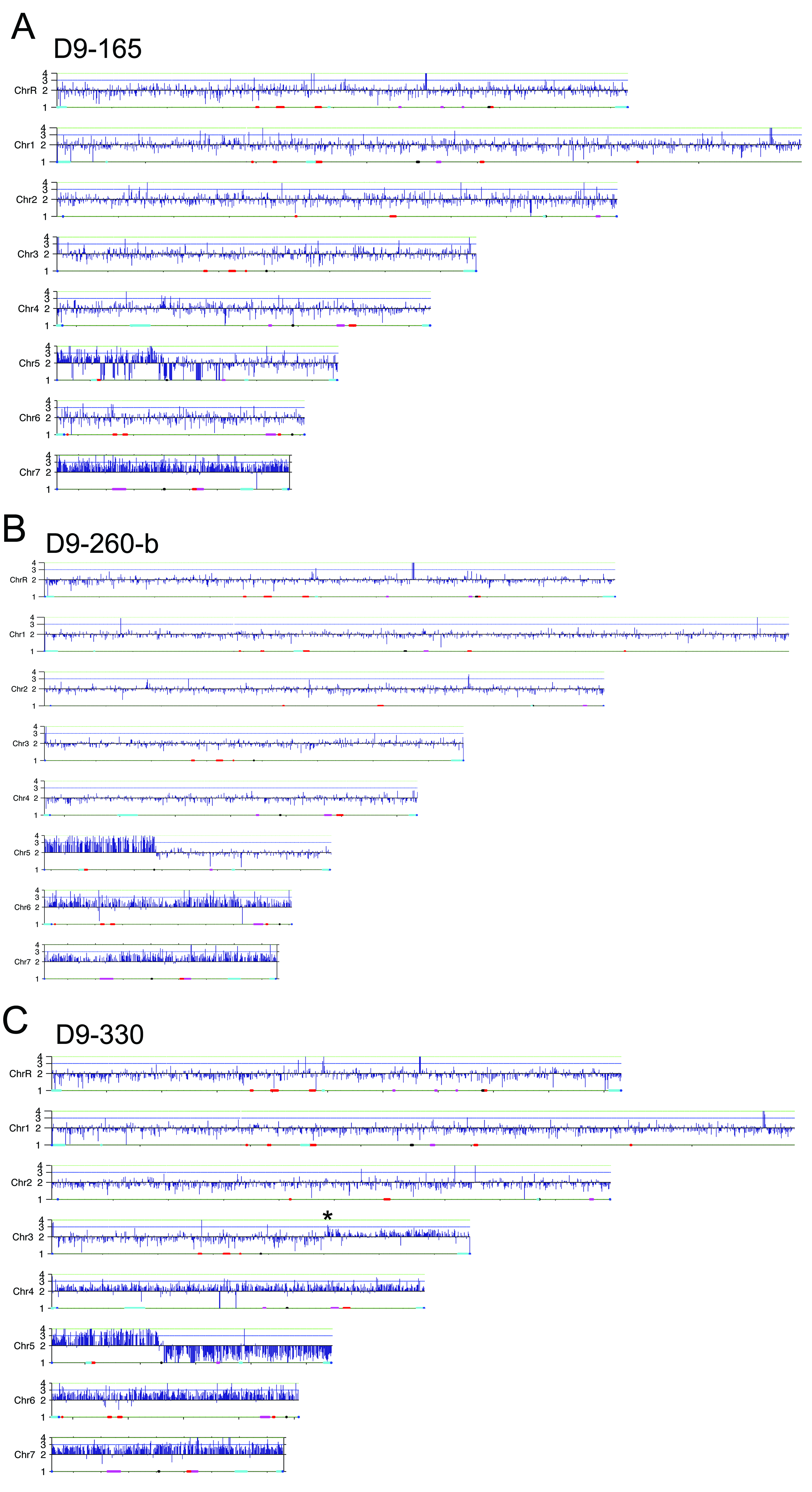

Supplement: Figure S6 — Comparative genome hybridization of D9. (A) D9-165 contains i(5L) and Chr7 trisomy, (B) in clone D9-260-b the i(5L) with additional Chr5L copies and Chr6 and Chr7 trisomies, while (C) D9-330 has i(5L), trisomy of Chr4, Chr6, and Chr7, Chr5 monosomy, and a small increase in copy number on the right arm of Chr3 (at the same breakpoint found on the i(5L)-3R chromosome, asterisk). (5.42 MB TIF) [file pgen.1000705.s006.tif]

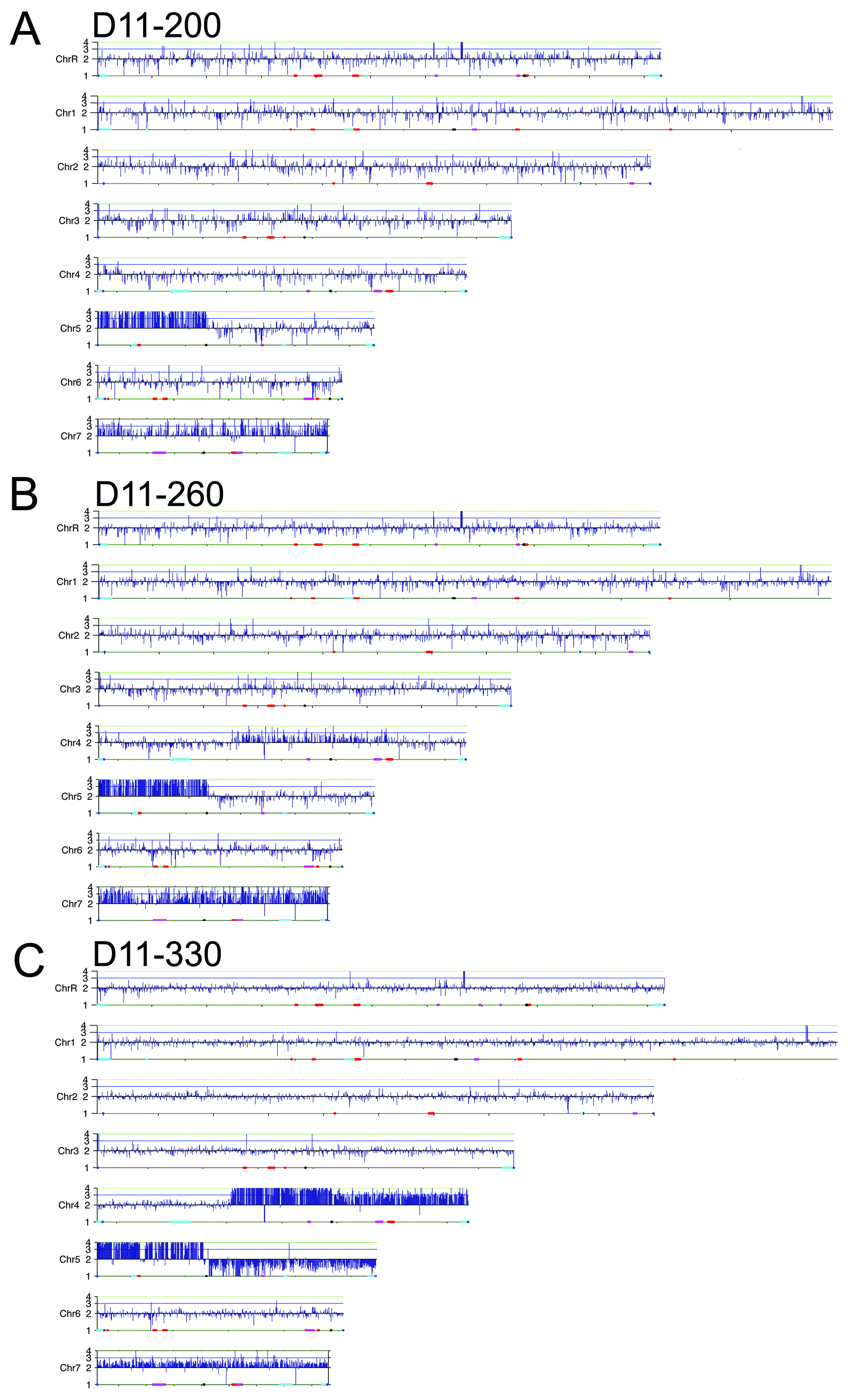

Supplement: Figure S7 — Comparative genome hybridization of aneuploid D11 populations. All 3 populations, (A) D11-200, (B) D11-260, and (C) D11-330 have the i(5L) and are trisomic for Chr7. These populations also have increased copy numbers of i(5L), ∼2 copies per cell, based on Log2 values (Average Log2 values of Chr5L from two arrays 1.04+/−0.01, compared to the Average Log2 value of trisomic Chr7 from these same arrays of 0.41+/−0.09). D11-260 has a segmental trisomy on Chr4 that either gets further amplified or becomes fixed in the population, such that by generation 330 the region is highly anueuploid: it includes three copies of the entire right arm of Chr4 and at CEN4 there is a transition to more than four copies of part of the left arm of Chr4. This amplified region includes NCP1, which encodes NADPH-cytochrome P450 reductase and is a co-factor of Erg11p. Finally, in population D11-300, Chr5R is monosomic. (5.51 MB TIF) [file pgen.1000705.s007.tif]

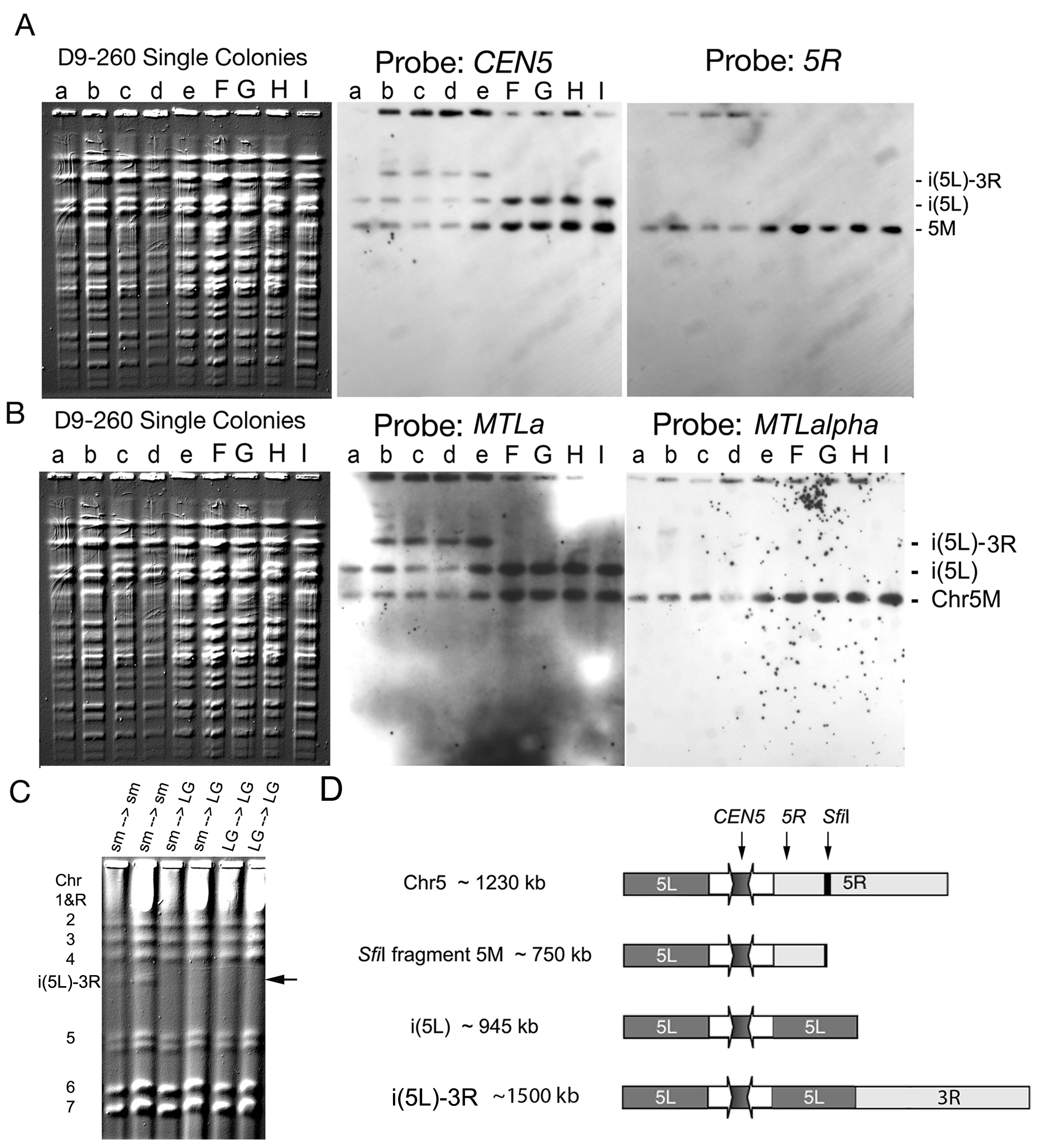

Supplement: Figure S8 — Further characterization of the i(5L)-3R SNC. (A) CHEF gel analysis of SfiI digested chromosomal DNA followed by Southern hybridization to a CEN5 probe identified three bands (diagramed in D): the centromere-containing 5M band from intact Chr5 (∼750 kb), the full-size i(5L) (∼945 kb), and the full-size SNC (∼1.5 Mb). Hybridization with a probe to the right arm of Chr5 only detects the ∼750 kb 5M band (right panel). (B) The i(5L)-3R SNC and the independent i(5L) in D9-260 colonies both hybridize to the MTLa allele, and not to the MTLα allele. The same SfiI-digested CHEF/Southern from (A) was probed with MTLa (left panel) and with MTLα (right panel). Bands representing i(5L)-3R, independent i(5L), and the SfiI-digested Chr5M are indicated. (C) The small colony phenotype of D9-260 is due to the presence of i(5L)-3R. Ethidium Bromide-stained CHEF gel of single colonies derived from either a small (sm) or large (LG) colony from the D9-260-b population. CHEF gel plugs were prepared from a small colony that gave rise to another small colony (sm→sm), a small colony that gave rise to a large colony (sm→LG), or a large colony that gave rise to another large colony (LG→LG). Only small colonies maintained the i(5L)-3R SNC, while large colonies derived from the small colonies had lost the chromosome. (D) Diagram of a full-length Chr5 homolog depicting the location of the CEN5 probe, the Chr5R probe and the sole SfiI restriction site, the digested ∼750 kb fragment from Chr5, the i(5L) lacking any SfiI sites and the ∼1.5 Mb SNC composed of 2 arms of Chr5L, one CEN5, and part of Chr3R, which also has no SfiI sites. (3.46 MB TIF) [file pgen.1000705.s008.tif]
