# Supplementary material for: Acquisition of Aneuploidy Provides Increased Fitness during the Evolution of Antifungal Drug Resistance
Source: PLoS Genet. 2009 Oct 30;5(10):e1000705. doi: 10.1371/journal.pgen.1000705 (PMC2760147; doi:10.1371/journal.pgen.1000705)
Supplement: Table S1 — Probes and primers used in this study. (0.13 MB DOCX) [file pgen.1000705.s009.docx]

**Table S1**: Probes and Primers used in this study

| **Probe** | **Chromosome position (Assembly 21)** | **Primer ID** | **Sequence (5’ – 3’)** |
| --- | --- | --- | --- |
| *CDR1* | chr3:1146155 to 1150660 | 2125-F | CAAAGACAAACTACTGCTGAT |
|  |  | 2126-R | CCCAGTATAAATAACCAT |
| *CDR2* | chr3:1047997 to 1052496 | 2114-F | TGGTTTGCATTTATTGCTGG |
|  |  | 2115-R | TAACCACCAGCAGCTTCG |
| *CEN3* | chr3:823335 to 826483 | 2436-F | ctagtcatgtcaggaggatg |
|  |  | 2437-R | CAATCAGACTTTGCAACGCC |
| 5L Telomere | chr5:3999 to 4808 | 2203-F | CCCATCGACCACCCATTA |
|  |  | 2204-R | GGCGTCGATTTTGGAATG |
| 5L *MTLa1* | chr5:394488 to 393526 | 3144-F | AAGCGACCAGATGGATGATG |
|  |  | 3145-R | CCCTTTCTCTTCGATTAGGC |
| 5L *MTLalpha1* | chr5:77072 to 77653 | 3146-F | CCAGATCTTCCTGTTGCTAG |
|  |  | 3147-R | GTTCTCGTTGGTGTTCCGGA |
| *CEN5* | chr5:468716 to 471745 | 2010-F | TACTTCTGGTCAACGAGGCT |
|  |  | 2011-R | GACTGACATCCGTACTATCG |
| 5R-IR | chr5:474103 to 475124 | 2851-F | AATAGGGCTCAAAGGCTG |
|  |  | 2854-R | CTAACGAATCCCGCGCAAAC |
| 5R | chr5:1036689 to 1038089 | 2076-F | TTTCGGGGAGAAGGCAAT |
|  |  | 2077-R | ATGGCTTGACAGGGTCCA |
| *HIS1* | chr5:1154134 to 1153238 | 2021-F | ttcttgtgccttgggtgttg |
|  |  | 2022-R | tgcttggatggattttgggg |
| 6L | chr6:254562 to 266027 | 2207-F | GTTCCCTCCACGGTGTTG |
|  |  | 2208-R | TAGCCACTCCATTTGCCG |
| 6L | chr6:433257 to 433516 | 1534-F | GAGGAGATAGTTGTTGCTGTC |
|  |  | 2336-R | GCACAGTAGTCAATGCCTAC |
| 6R | chr6:1021078 to 1021333 | 2114-F | TCTGTCGCCTGAGCTGTTTA |
|  |  | 2404-R | GGGTATTTTGGGTCGTTTGG |
| *CEN7* | chr7:425812 to 428712 | 2441-F | AGGATCTATTGACGAGGGAGAAT |
|  |  | 2442-R | GCATTAATCCCCAATTATCAACG |
|  |  |  |  |
| ***NAT1* Tagging Primers** |  |  |  |
| Amplify *NAT1* construct from pMG2021 |  | oLC596 | GAGAATCGAAGAAGAATTAGGT  TCTGAAGCTATCTACGCTGGTA  AAGATTTCCAAAAGGCTTCTCA  ATTGGGTGGTGGTTCTAAAGGT  GAAGAATTAT |
| Amplify *NAT1* construct from pMG2021 |  | oLC597 | CAGCTCAGTGATTAAGAGTAAAGA  TGGGTAAAAAATTATCATTTAATT  AGTTCATATATTCAAGATGTTCCG  TAAAACGACGGCCAGTGAATTC |
| Check Transformants |  | oLC598 | CACAGATCCGGTGAAACCG |
| Check Transformants |  | oLC599 | GTAAATAGACGTTAGCATCG |
| Check Transformants |  | oLC600 | CCTTCAAACTTGACTTCAGC |
| Check Transformants |  | oLC601 | CAGCACCTCTTTCTCTGG |
